# Enhancing stability control of inverted pendulum using Takagi–Sugeno fuzzy model with disturbance rejection and input–output constraints

**DOI:** 10.1038/s41598-023-41258-3

**Published:** 2023-09-02

**Authors:** Thi-Van-Anh Nguyen, Bao-Trung Dong, Ngoc-Tam BUI

**Affiliations:** 1 Hanoi University of Science and Technology, Hanoi, 11615 Vietnam; 2 Shibaura Institute of Technology, Saitama, 337-8570 Japan

**Keywords:** Electrical and electronic engineering, Mechanical engineering

## Abstract

The Takagi–Sugeno (T–S) fuzzy model is a versatile approach widely used in system control, often in combination with other strategies. This paper addresses key control challenges linked to the T–S system and presents important considerations to ensure its successful application using the Lyapunov theorem. One crucial aspect is determining the optimal number of premise variables and selecting accurate fuzzy rules for the T–S model. Additionally, the theorem based on Linear Matrix Inequality (LMI) is developed to enable effective disturbance rejection. To enhance stability control, constraints are imposed on the output angle and control input of a rotary inverted pendulum (RIP). By integrating T–S fuzzy control, disturbance rejection, and input/output constraints, robust stability in controlling the RIP is achieved. Extensive simulations are performed to showcase the efficiency of the suggested method, and the simulation results are thoroughly discussed and analyzed to verify the efficacy of the control method.

## Introduction

The inverted pendulum^1,2^ is a well-known and extensively studied control problem that has attracted significant research attention due to its inherent instability and practical applications in various fields. The rotary inverted pendulum (RIP)^3,4^, a fascinating and extensively studied control problem, presents unique challenges due to its rotational nature. Unlike the traditional inverted pendulum, which moves along a horizontal track, the RIP involves a pendulum attached to a rotating base. The pendulum’s balance must be maintained by controlling the rotational speed of the base. This system exhibits complex dynamics, involving both rotational and translational motion, as well as the interplay between centrifugal forces, inertia, and gravitational effects. Achieving stability and control in the RIP requires sophisticated strategies that account for the intricate coupling between the pendulum and the rotational base. By comprehensively understanding the kinematics and mechanics of the RIP, researchers can develop robust control approaches that ensure precise and stable operation in various applications. This system is classified as an underactuated system^5–7^ since the number of control inputs is fewer than the number of degrees of freedom. Consequently, it requires a sophisticated control strategy to achieve stability.

Over the years, numerous control approaches have been proposed and investigated to tackle the inverted pendulum control problem, each aiming to overcome the inherent instability and achieve robust stabilization. These include classical control techniques, such as PID control^8^, as well as modern control methodologies, such as adaptive finite-time command-filtered backstepping sliding mode control^9^, indirect adaptive fuzzy model predictive control^10^ and adaptive neural network control^11^. While these methods have shown varying degrees of success, they often face limitations in terms of robustness, adaptability to nonlinearities, and handling uncertainties, which are critical factors in achieving stable and efficient control of the inverted pendulum system. The challenges encountered in the control of the RIP are similar to those faced in the traditional inverted pendulum system. Robustness, adaptability to nonlinearities, and handling uncertainties are key concerns. Robustness ensures stable performance in the face of disturbances or parameter variations. Nonlinearities, such as friction or nonlinear dynamics, significantly impact the system’s behavior, making the design of effective control strategies more complex. Additionally, uncertainties in system parameters or external factors further complicate the control problem, as accurate modeling and prediction become more challenging. Successfully addressing these challenges in the context of the RIP requires robust control techniques capable of handling nonlinear dynamics and effectively accounting for uncertainties. By developing advanced control strategies that address these issues, our goal is to achieve stable and efficient control of the RIP system.

Fuzzy logic controllers have emerged as a powerful and flexible tool within the field of control theory, revolutionizing the way complex and uncertain systems are controlled^12,13^. One particularly promising and widely studied control methodology for addressing the challenges posed by the inverted pendulum system is the use of Takagi–Sugeno (T–S) fuzzy control^14,15^. The T–S fuzzy control approach^16–18^ combines the flexibility of fuzzy logic with the power of local linear models to provide a versatile and adaptive control framework. By dividing the system into multiple local linear models associated with fuzzy if-then rules, T–S fuzzy control offers a systematic means of capturing the nonlinearity and uncertainty present in the inverted pendulum dynamics. This allows for precise control under various operating conditions and enhances the system’s robustness and adaptability^19,20^. In the T–S fuzzy model, an important aspect is the choice of the appropriate premise variables and the decision regarding the number of fuzzy rules. The choice of premise variables involves identifying the key factors that influence the system dynamics and selecting appropriate variables to represent them. This requires a thorough understanding of the system behavior and its underlying mechanisms. Additionally, determining the number of fuzzy rules involves finding the right balance between model complexity and computational efficiency. It is crucial to have an adequate number of rules to capture the system’s nonlinearities and uncertainties effectively, while avoiding excessive computational burden. Careful consideration and analysis are required to ensure the optimal selection of premise variables and the appropriate number of fuzzy rules in the T–S fuzzy model. Moreover, researchers have developed innovative control strategies based on T–S fuzzy control, such as the parallel distributed compensation (PDC) controller^21^, which enables distributed control and effective handling of system complexity. The stability and performance of T–S fuzzy control systems are often analyzed and ensured using mathematical techniques like Linear Matrix Inequalities (LMI)^22–24^, which provide a powerful framework for stability analysis and controller design, guaranteeing robust stability and performance. For example, the researchers have demonstrated the effectiveness of T–S fuzzy control by representing the system using a T–S fuzzy model, applying the PDC control method, and verifying the stability using LMI-based analysis^25^. By integrating T–S fuzzy control, PDC controller, and LMI techniques, a comprehensive and effective approach is established to address the stability control problem in inverted pendulum systems, contributing to advancements in control methodologies and opening up new possibilities for practical applications.

In the realm of RIP control, the rejection of disturbances is a critical challenge. Disturbances, originating from external forces, environmental factors, or changes in operating conditions, can disrupt the equilibrium of the pendulum. To address this problem, researchers have explored robust, adaptive, and intelligent control strategies, including the use of the $H \infty$ control method^26^. In this work, we leverage the power of the $H \infty$ method within the fuzzy control context to design a controller that not only provides robust stability but also effectively rejects disturbances. By combining the flexibility and adaptability of T–S fuzzy control with the disturbance rejection capabilities of $H \infty$, we aim to achieve precise and stable control of the RIP system.

In addition to addressing the challenge of disturbance rejection, the present work also considers the important aspect of handling input and output constraints in the control of the RIP system. In practice, it is often necessary to impose limitations on the pendulum angle position to ensure safe operation and prevent the pendulum from deviating beyond a certain range. Similarly, constraints on the control input, such as voltage or torque, may be required to comply with physical limitations or operational constraints. To effectively handle these constraints, constraint-based methodologies are integrated within the T–S fuzzy control framework. By incorporating constraints on the pendulum angle output and the control input, the system operates within predefined limits, enhancing safety and stability. The proposed technique, such as constrained optimization, generates control signals that satisfy the constraints while achieving optimal performance. This comprehensive approach to handling input and output constraints further enhances the practical applicability and effectiveness of the control strategy for the RIP system presented in this paper.

The main contribution of this paper is the development of a comprehensive control strategy for the stability and efficient control of the rotary inverted pendulum system.

- The appropriate selection of variables premises in the T–S fuzzy model for RIP system.
- The development of a control strategy that integrates T–S fuzzy control and the $H \infty$ method, resulting in a more stable and robust control system with the disturbance rejection capabilities for the RIP.
- Effectively handling input and output constraints in the control of the RIP, ensuring that the pendulum angle position and control input remain within predefined limits to enhance safety and stability.
- Constrained optimization techniques are applied to generate control signals that satisfy imposed constraints and optimize the performance of the RIP system, ensuring efficient control while respecting limitations and operational constraints.
- Providing a comprehensive and effective control strategy with practical applications and opportunities for further research and development.

The next section provides an introduction to the T–S model, which will be specifically applied to the control of a RIP. In this section, the appropriate quantity of fuzzy rules for controlling the pendulum will be determined. Following that, the main results section presents some theorems that address stability, disturbance rejection, and input-output constraints within the T–S model framework. The simulation results of these cases will then be showcased to validate the efficacy of the proposed control approach for the RIP. Finally, the conclusion will summarize the findings and highlight the contributions of the study.

## T–S fuzzy model

The T–S fuzzy model utilized in this study is based on the principles of fuzzy logic. Unlike Mamdani’s method, Sugeno proposed a simplified approach for analyzing and controlling nonlinear systems by representing them as a collection of model subsystems. Each subsystem is characterized by an IF-THEN rule that establishes a relationship between the input and output signals of the system. T–S method relies on locally linearizing the nonlinear systems, providing a practical framework for analysis and control. It is important to note that this article exclusively focuses on continuous-time systems. Therefore, the equations, calculation formulas, and theorems presented in this study are specifically applicable to continuous-time systems.

The T–S fuzzy model for continuous-time systems is described by the following equation:

1 $$\begin{aligned} \hspace{5cm}{\left\{ \begin{array}{ll} \varvec{\dot{x}}(t) = A\varvec{x}(t) + B\varvec{u}(t)\\ \varvec{y}(t) = C\varvec{x}(t) \end{array}\right. } \end{aligned}$$

where $\varvec{x}(t)$ is state vector, $\varvec{x}(t)$ $\in R^n$; $\varvec{y}(t)$ is output vector, $\varvec{y}(t)$ $\in R^q$; $\varvec{u}(t)$ is input vector, $\varvec{u}(t)$ $\in R^m$; *A*, *B*, *C* are matrix, $A \in R^{n\times n}$, $B \in R^{n\times m}$, $C \in R^{q\times n}$. In the context of the T–S fuzzy system, the state variables of the system, denoted by $\sigma (t)$, play a crucial role as the premise variables. These variables describe the dynamics behavior of the system and serve as the basis for the fuzzy rule-based control approach. These variables are represented as:

2 $$\begin{aligned} \hspace{5cm}\sigma (t) = f(\varvec{x}_1,\varvec{x}_2,...,\varvec{x}_n) \end{aligned}$$

with $\varvec{x}(t) = [\varvec{x}_1(t)\ \varvec{x}_2(t)\ \ldots \ \varvec{x}_n(t)]^T$. It is important to note that $\sigma (t)$ can be described as a set of component variables in vector form, represented as follows:                                     

3 $$\begin{aligned} \hspace{5cm}\sigma (t) = [\sigma _1(t) \ \sigma _2(t)\ \ldots \sigma _p(t)].\end{aligned}$$

The number of rules in the symbolic model is denoted as *r* and can be calculated by ${r} = 2^p$. In this control method, a crucial concept is the membership function, which plays a key role in modeling the system’s states. However, it is important to note that this method involves two different types of membership functions. These types represent the equation of state and are expressed using a membership function in the following form:

4 $$\begin{aligned}{} & {} \varvec{\dot{x}}(t) = \dfrac{\sum \limits _{i=1}^{r}w_{i}(\sigma (t))\{A_{i}\varvec{x}(t)+B_{i}\varvec{u}(t)\}}{\sum \limits _{i=1}^ {r}w_{i}(\sigma (t))} \end{aligned}$$

5 $$\begin{aligned}{} & {} \varvec{\dot{x}}(t) = \sum \limits _{i=1}^{r}h_{i}(\sigma (t))\{A_{i}\varvec{x}(t) + B_{i}\varvec{u}(t)\}\end{aligned}$$

where $w_{i}(\sigma (t)) = \prod \limits _{j=1}^{p}M_{ij}(\sigma _{j}(t))$ and $h_{i}(\sigma (t)) = \dfrac{w_{i}(\sigma (t))}{\sum \limits _{i=1}^{r}w_{i}(\sigma (t))}$ for all *t*. $M_{ij}(\sigma _{j}(t))$ is the grade of $\sigma _{j}(t)$. In the T–S fuzzy method, an important property known as the convex sum property plays a crucial role in combining the outputs of individual fuzzy rules to obtain the overall system response. This property ensures that the overall fuzzy output is obtained through a convex combination of the outputs of activated rules, weighted by their respective degrees of activation. By employing the convex sum property, the T–S fuzzy method achieves smooth transitions between different fuzzy sets and allows for adaptive and flexible control of nonlinear systems. For all *t*, denote that:

6 $$\begin{aligned} \hspace{5cm}{\left\{ \begin{array}{ll} \sum \limits _{i=1}^{r}h_{i}(\sigma (t)) = 1,\\ h_{i}(\sigma (t)) \ge 0. \end{array}\right. } \end{aligned}$$

Concluding the section on the T–S fuzzy model, it is evident that this approach offers a versatile and adaptive control framework for precise control of nonlinear systems. By employing fuzzy rules, membership functions, premise variables, and state equations, the T–S fuzzy model effectively captures the complexities of system dynamics and provides a systematic control design methodology. With this foundation in place, the subsequent section will focus on the application of the T–S fuzzy model to the RIP system. This application aims to address the unique challenges posed by the rotary inverted pendulum and develop a robust control strategy that ensures stability, robustness, and optimal performance.

## Application of T–S fuzzy model to the rotary inverted pendulum system

**Figure 1:**
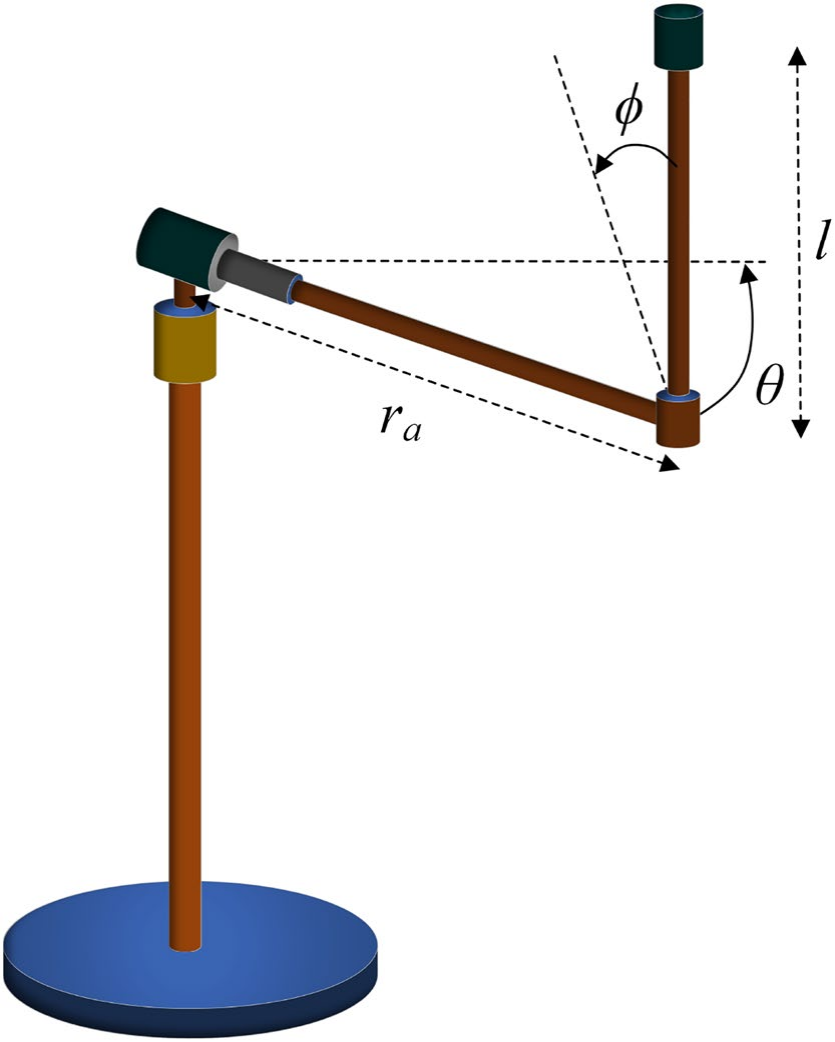
The rotary inverted pendulum system.

In this section, the application of the T–S fuzzy model to the RIP system is presented. The section begins with an overview of the kinematic modeling of the RIP system, followed by the transformation of this model into T–S fuzzy equations. Furthermore, a discussion is provided on how to choose the premise variable and the quantity of fuzzy rules for the RIP system. The RIP system comprises a servo motor system and two pendulum rods. The pendulum arm rod is characterized by a length denoted as $r_a$, whereas the pendulum rod has a length of *l* and a mass of *m*, see Fig. 1. Table 1 presents the parameters of the RIP system^27^.

**Table 1:** The rotary inverted pendulum parameters.

Gravity $(ms^{-2})$	*g*	9.81
Mass of the pendulum rod (*kg*)	*m*	0.125
Length of the pendulum rod (*m*)	*l*	0.335
Length of the pendulum arm (*m*)	$r_a$	0.215
Equivalent moment of inertia of the pendulum arm and gears $(kgm^2)$	$J_{eq}$	$3.5842\times 10^{-3}$
Moment of inertia of the motor rotor $(kgm^2)$	$J_m$	$3.87\times 10^{-7}$
Friction coefficient of the pendulum arm $(Nmsrad^{-1})$	$B_a$	0.004
Friction coefficient of the pendulum rod $(Nmsrad^{-1})$	$B_r$	0.0095
Torque constant $(NmA^{-1})$	$K_t$	$7.67\times 10^{-3}$
Back EMF constant $(Vsrad^{-1})$	$K_v$	$7.67\times 10^{-3}$
Motor armature resistance $(\Omega )$	*R*	2.6
Gearbox ratio	$K_g$	70
Gearbox efficiency	$n_g$	0.9
Motor efficiency	$n_m$	0.69

The equations of state for the RIP system can be represented as

7 $$\begin{aligned} \ddot{\phi }&=3(s_{4}s_{5}sin{\phi }+ s_{1}s_{4}sin^3{\phi }- s_{3}s_{5}\dot{\phi } - V_{m}s_{2}s_{7}cos{\phi } + s_{2}s_{6}\dot{\theta }cos{\phi } - s_{2}^2\dot{\phi }^2cos{\phi }sin{\phi } +2s_{1}s_{2}\dot{\phi }\dot{\theta }cos^2{\phi }sin{\phi }\nonumber \\&\quad + s_{1}^2\dot{\theta }^2cos{\phi }sin^3{\phi } +s_{1}s_{5}\dot{\theta }^2cos{\phi }sin{\phi } - s_{1}s_{3}\dot{\phi }sin^2{\phi }) (4s_{1}s_{5} - 3s_{2}^2cos^2{\phi } + 4s_{1}^{2}sin^2{\phi })^{-1}, \end{aligned}$$

8 $$\begin{aligned} \ddot{\theta }&= -(4s_{1}s_{6}\dot{\theta } - 3s_{2}s_{3}\dot{\phi }cos{\phi }-4s_{1}s_{2}\dot{\phi }^2sin{\phi }-4V_{m}s_{1}s_{7} +8s_{1}^{2}\dot{\phi }\dot{\theta }cos{\phi }sin{\phi }+3s_{1}s_{2}\dot{\theta }^{2}cos^2{\phi }sin{\phi }\nonumber \\&\quad +3s_{2}s_{4}cos{\phi }sin{\phi })(4s_{1}s_{5} - 3s_{2}^2cos^2{\phi } + 4s_{1}^{2}sin^2{\phi })^{-1} \end{aligned}$$

where the values of $s_{1},s_2,s_3,s_4,s_5,s_6,s_7$ are determined by the following formulas:

$$\begin{aligned} s_1 = \dfrac{ml^2}{4},\ s_2 = \dfrac{mlr_a}{2},\ s_3 = B_r,\ s_4 = \dfrac{mgl}{2},\ s_5 = J_{eq} + mr^2_a + n_gK_g^2J_m,\ \\ \nonumber s_6 = B_{a} + \dfrac{n_mn_gK_tK_vK_g^2}{R},\ s_7 = \dfrac{n_mn_gK_tK_g}{R}, \end{aligned}$$

and $V_m$ is voltage input. Since the inception of the Takagi–Sugeno fuzzy system modeling method, there has been no established standard for constructing premise variables that are the most suitable and optimal for the model. Most axiomatic variables are developed based on individual experience. The absence of a rigid framework in the selection of axiomatic variables for model construction has provided flexibility, which is also a notable advantage of the Takagi–Sugeno fuzzy method. However, the absence of specific binding rules in building the Takagi–Sugeno fuzzy model can sometimes lead to challenging cases during system simulation. This article aims to present examples of errors encountered in the model-building process, along with their causes and simpler, more optimal solutions. For instance, considering the equation representing the state of the RIP system, Eq. (1) with $\varvec{x}(t) = [\phi (t)\ \theta (t)\ \dot{\phi }(t)\ \dot{\theta }(t)]^T$, it can be expressed as follows:                                     

$$\begin{aligned} A = \begin{bmatrix} 0&{} 0&{} 1&{} 0\\ 0&{} 0&{} 0&{} 1\\ 0&{} 0&{} \alpha _{1}&{} \beta _{1}\\ 0&{} 0&{} \gamma _{1}&{} \omega _{1} \end{bmatrix}, \hspace{1cm} B = \begin{bmatrix} 0\\ 0\\ \kappa \\ \varepsilon \end{bmatrix} \end{aligned}$$

where

$$\begin{aligned}{}&\alpha _{1} =\ 3(-s_{2}^2\dot{\phi }cos{\phi }sin{\phi } -s_{1}s_{3}sin^2{\phi }+\dfrac{s_{4}s_{5}sin{\phi }}{\dot{\phi }} -s_{3}s_{5}+\dfrac{s_{1}s_{4}sin^3{\phi }}{\dot{\phi }})(4s_{1}s_{5} - 3s_{2}^2cos^2{\phi } + 4s_{1}^{2}sin^2{\phi })^{-1},\\&\beta _{1} =\ 3(s_2s_6cos{\phi }+ s_{1}^2\dot{\theta }cos{\phi }sin^3{\phi } + s_{1}s_{5}\dot{\theta }cos{\phi }sin{\phi } + 2s_{1}s_{2}\dot{\phi }cos^2{\phi }sin{\phi }) (4s_{1}s_{5} - 3s_{2}^2cos^2{\phi } + 4s_{1}^{2}sin^2{\phi })^{-1},\\&\gamma _{1} =\ (3s_2s_3cos{\phi }+4s_1s_2\dot{\phi }sin{\phi } - 3\dfrac{s_2s_4cos{\phi }sin{\phi }}{\dot{\phi }}) (4s_{1}s_{5} - 3s_{2}^2cos^2{\phi } + 4s_{1}^{2}sin^2{\phi })^{-1},\\&\omega _{1} = -(4s_1s_6 +3s_{1}s_{2}\dot{\theta }cos^2{\phi }sin{\phi } + 8s_{1}^2\dot{\phi }cos{\phi }sin{\phi }) (4s_{1}s_{5} - 3s_{2}^2cos^2{\phi } + 4s_{1}^{2}sin^2{\phi })^{-1},\\&\kappa =\ \dfrac{-3s_2s_7cos{\phi }}{4s_{1}s_{5} - 3s_{2}^2cos^2{\phi } + 4s_{1}^{2}sin^2{\phi }}, \hspace{0.3cm}\varepsilon =\ \dfrac{4s_1s7}{4s_{1}s_{5} - 3s_{2}^2cos^2{\phi } + 4s_{1}^{2}sin^2{\phi }}. \end{aligned}$$

By utilizing the equation of state, a T–S fuzzy model consisting of 64 rules can be derived as shown below:

$$\begin{aligned}{}&\sigma _{1}(t) =\ \dfrac{1}{4s_{1}s_{5} - 3s_{2}^2cos^2{\phi } + 4s_{1}^{2}sin^2{\phi }}, \hspace{0.3cm}\sigma _{2}(t) =\ 3(\dfrac{s_{4}s_{5}sin{\phi }+ s_{1}s_{4}sin^3{\phi }}{\dot{\phi }}- s_{3}s_{5} -s_{2}^2\dot{\phi }cos{\phi }sin{\phi }-s_{1}s_{3}sin^2{\phi }),\\&\sigma _{3}(t) =\ 3(s_2s_6cos{\phi }+s_{1}^2\dot{\theta }cos{\phi }sin^3{\phi } +s_{1}s_{5}\dot{\theta }cos{\phi }sin{\phi } + 2s_{1}s_{2}\dot{\phi }cos^2{\phi }sin{\phi }),\hspace{0.3cm}\sigma _{4}(t) =\ -3s_2s_7cos{\phi },\\&\sigma _{5}(t) =\ 3s_2s_3cos{\phi }+4s_1s_2\dot{\phi }sin{\phi }-3\dfrac{s_2s_4cos{\phi }sin{\phi }}{\dot{\phi }},\hspace{0.3cm}\sigma _{6}(t) =\ -(4s_1s_6 +3s_{1}s_{2}\dot{\theta }cos^2{\phi }sin{\phi }+8s_{1}^2\dot{\phi }cos{\phi }sin{\phi }).\\ \end{aligned}$$

Subsequently, the matrices *A* and *B* are transformed as follows:

$$\begin{aligned} A = \begin{bmatrix} 0&{} 0&{} 1&{} 0\\ 0&{} 0&{} 0&{} 1\\ 0&{} 0&{} \sigma _{1}(t)\sigma _{2}(t) &{} \sigma _{1}(t)\sigma _{3}(t)\\ 0&{} 0&{} \sigma _{1}(t)\sigma _{5}(t)&{} \sigma _{1}(t)\sigma _{6}(t) \end{bmatrix}, \hspace{1cm} B = \begin{bmatrix} 0\\ 0\\ \sigma _{1}(t)\sigma _{4}(t)\\ 4s_1s_7\sigma _1(t) \end{bmatrix}. \end{aligned}$$

Consider the following:

Let $D = R^4$ be the state space of an RIP system, with a state vector represented as $\varvec{x}(t) = [\phi (t)\ \theta (t)\ \dot{\phi }(t)\ \dot{\theta }(t)]^T$. It is evident that the functions $\sigma _1(t), \sigma _3(t), \sigma _4(t), \sigma _6(t)$ are continuous functions in *D*, $\forall \varvec{x}(t) \in D$. Now, let’s consider a set of state space vectors in the form of $\varvec{x}_{\varvec{itr}\mathfrak {j}} = [\phi _{\mathfrak {j}} \ \theta _{\mathfrak {j}} \ \dot{\phi }_{\mathfrak {j}} \ \dot{\theta }_{\mathfrak {j}}]^{T}$, where it is assumed that $\dot{\phi }_{\mathfrak {j}} = 0, \ \forall {\mathfrak {j}} \in N$. Specifically, we have the following limits:

$$\begin{aligned}{} & {} \lim _{{\dot{\phi } \rightarrow 0^+}}\left( \frac{{s_{4}s_{5}\sin {\phi } + s_{1}s_{4}\sin ^3{\phi }}}{{\dot{\phi }}}\right) = +\infty , \forall \phi \ne 0; \\{} & {} \lim _{{\dot{\phi } \rightarrow 0^-}}\left( \frac{{s_{4}s_{5}\sin {\phi } + s_{1}s_{4}\sin ^3{\phi }}}{{\dot{\phi }}}\right) = -\infty , \forall \phi \ne 0. \end{aligned}$$

Therefore, the function $\sigma _2(t)$ experiences an interruption at $\dot{\phi } = 0$, for all $\phi \ne 0$. Furthermore, it should be noted that the left limit of $\sigma _2(t)$ as $\dot{\phi }$ approaches 0 from the negative side is different from the right limit of $\sigma _2(t)$ as $\dot{\phi }$ approaches 0 from the positive side. In this case, it is apparent that achieving continuity for the function $\sigma _2(t)$ is not feasible. It is also observed that $\sigma _5(t)$ is an interrupted function at $\dot{\phi } = 0$ for all $\phi$ $\ne 0$. As a result, the system described by the 64 rules, as given in Eqs. (7) and (8), exhibits an interruption function at $\dot{\phi } = 0$ for all $\phi$ $\ne 0$.

Hence, within the RIP system model consisting of 64 rules, there exists a set of state vectors that can induce interruptions in the system. This set of state vectors is of the form: $\varvec{x}_{\varvec{itr}\mathfrak {j}} = [\phi _\mathfrak {j} \ \theta _\mathfrak {j} \ 0 \ \dot{\theta }_\mathfrak {j}]^T$, $\forall \ \phi \ne 0$. To ensure effective system control, it becomes necessary to apply stability criteria to the existing model. However, it is important to acknowledge that each stability criterion imposes specific constraints. In the context of utilizing the Takagi–Sugeno fuzzy model for control, the direct Lyapunov method is often employed as it provides a straightforward approach backed by available tools and theorems.

The Lyapunov function in the system is represented by the equation:

9 $$\begin{aligned} \hspace{5cm}V(\varvec{x}(t)) = \varvec{x}^T(t)P\varvec{x}(t). \end{aligned}$$

To utilize Lyapunov’s theorem for continuous time for controlling a stable operating system, it is necessary for the system to satisfy specific conditions:

10 $$\begin{aligned} \hspace{5cm}{\left\{ \begin{array}{ll} V(\varvec{x}(t)) = 0\ \text {while}\ \varvec{x}(t) = 0\\ V(\varvec{x}(t))> 0 , \ \forall \varvec{x}(t) \ne 0\\ \exists \ \dot{V}(\varvec{x})<0,\ \forall t >0. \end{array}\right. } \end{aligned}$$

In the direct Lyapunov condition, it is necessary for the Lyapunov function to be differentiable. However, the T–S fuzzy model, consisting of 64 rules, is a discontinuous model. Consequently, the direct Lyapunov theorem does not directly apply to this model. Applying Lyapunov’s theorem directly to the T–S fuzzy model described by the 64 rules mentioned above would lead to incorrect results. This article aims to propose a straightforward solution utilizing the available mathematical tools. Therefore, it is crucial to carefully choose a function that satisfies the conditions of the theorem to be employed when selecting a rule. Now, we will proceed to construct the model described by 256 rules, unlike the previous model, fulfills all the conditions of Lyapunov’s theorem.

In this 256-rule model, slight changes will be made to the matrix *A*, while the matrix *B* remains unchanged.

$$\begin{aligned} A = \begin{bmatrix} 0&{} 0&{} 1&{} 0\\ 0&{} 0&{} 0&{} 1\\ \iota &{} 0&{} \alpha _2&{} \beta _2\\ \xi &{} 0&{} \gamma _2&{} \omega _2 \end{bmatrix} \end{aligned}$$

where

$$\begin{aligned}{}&\iota =\ 3\dfrac{s_{4}s_{5}sin{\phi }+ s_{1}s_{4}sin^3{\phi }}{\phi }(4s_{1}s_{5}- 3s_{2}^2cos^2{\phi } + 4s_{1}^{2}sin^2{\phi })^{-1},\\&\xi =\ -3\dfrac{s_2s_4cos{\phi }sin{\phi }}{\phi }(4s_{1}s_{5}- 3s_{2}^2cos^2{\phi } + 4s_{1}^{2}sin^2{\phi })^{-1},\\&\alpha _2 =\ 3(- s_{3}s_{5} - s_{2}^2\dot{\phi }cos{\phi }sin{\phi }-s_{1}s_{3}sin^2{\phi })(4s_{1}s_{5}- 3s_{2}^2cos^2{\phi } + 4s_{1}^{2}sin^2{\phi })^{-1},\\&\beta _2 =\ 3(s_2s_6cos{\phi }+ s_{1}^2\dot{\theta }cos{\phi }sin^3{\phi } + s_{1}s_{5}\dot{\theta }cos{\phi }sin{\phi } + 2s_{1}s_{2}\dot{\phi }cos^2{\phi }sin{\phi })(4s_{1}s_{5}- 3s_{2}^2cos^2{\phi } + 4s_{1}^{2}sin^2{\phi })^{-1},\\&\gamma _2 =\ (3s_2s_3cos{\phi }+4s_1s_2\dot{\phi }sin{\phi })(4s_{1}s_{5}- 3s_{2}^2cos^2{\phi } + 4s_{1}^{2}sin^2{\phi })^{-1},\\&\omega _2 =\ -(4s_1s_6 +3s_{1}s_{2}\dot{\theta }cos^2{\phi }sin{\phi } +8s_{1}^2\dot{\phi }cos{\phi }sin{\phi })(4s_{1}s_{5}- 3s_{2}^2cos^2{\phi } + 4s_{1}^{2}sin^2{\phi })^{-1}. \end{aligned}$$

To construct the new T–S model, select 8 premise variables in the form of:

$$\begin{aligned}{}&\sigma _1(t) =\ (4s_{1}s_{5} - 3s_{2}^2cos^2{\phi } + 4s_{1}^{2}sin^2{\phi })^{-1},\hspace{0.3cm} \sigma _2(t) =\ 3\dfrac{s_{4}s_{5}sin{\phi }+ s_{1}s_{4}sin^3{\phi }}{\phi },\\&\sigma _3(t) =\ -3 (s_{3}s_{5} + s_{2}^2\dot{\phi }cos{\phi }sin{\phi }+s_{1}s_{3}sin^2{\phi }),\hspace{0.3cm} \sigma _4(t) =\ -3s_2s_7cos{\phi },\\&\sigma _5(t) =\ 3(s_2s_6cos{\phi }+ s_{1}^2\dot{\theta }cos{\phi }sin^3{\phi } + s_{1}s_{5}\dot{\theta }cos{\phi }sin{\phi } + 2s_{1}s_{2}\dot{\phi }cos^2{\phi }sin{\phi }),\hspace{0.3cm} \sigma _6(t) =\ -3\dfrac{s_2s_4cos{\phi }sin{\phi }}{\phi },\\&\sigma _7(t) =\ 3s_2s_3cos{\phi }+4s_1s_2\dot{\phi }sin{\phi },\hspace{0.3cm} \sigma _8(t) =\ -(4s_1s_6 +3s_{1}s_{2}\dot{\theta }cos^2{\phi }sin{\phi } +8s_{1}^2\dot{\phi }cos{\phi }sin{\phi }). \end{aligned}$$

As a result, the matrix *A* is updated as follows:

$$\begin{aligned} A = \begin{bmatrix} 0&{} 0&{} 1&{} 0\\ 0&{} 0&{} 0&{} 1\\ \sigma _1(t)\sigma _2(t)&{} 0&{} \sigma _1(t)\sigma _3(t)&{} \sigma _1(t)\sigma _5(t)\\ \sigma _1(t)\sigma _6(t)&{} 0&{} \sigma _1(t)\sigma _7(t)&{} \sigma _1(t)\sigma _8(t) \end{bmatrix}. \end{aligned}$$

The Takagi–Sugeno fuzzy model described by these 256 rules exhibits a discontinuity at $\phi = 0$. However, it is possible to parameterize this discontinuity with a specific value, ensuring the uninterrupted nature of the Lyapunov function. Consequently, the direct application of the Lyapunov theorem becomes feasible.

## Main results

In this section, the key findings obtained from employing the T–S fuzzy model to the RIP system will be showcased. The section is organized into four subsections, each addressing a specific aspect of the system’s performance. These subsections encompass the stability theorem, disturbance rejection theorem, constraint output theorem, and constraint input theorem. Through an examination of these theorems, valuable insights into the stability, disturbance rejection capabilities, and handling of constraints by the T–S fuzzy model for the RIP system can be obtained. The subsequent exploration of each subsection will unveil the key findings and implications of the study.

### Stability analysis

Within the domain of T–S systems, the Parallel Distributed Compensation (PDC) method emerges as a popular choice for controlling system input. This method facilitates the construction of a controller comprising multiple parallel control modules, each correlated with a distinct fuzzy rule. Operating autonomously, these modules make localized control decisions grounded in the premise variables of their respective fuzzy rules. Through the aggregation of their outputs, an overarching control input is derived to govern the T–S system effectively. The control input of the system (1), formulated in the PDC form, can be represented by the following equation:

11 $$\begin{aligned} \hspace{5cm}\varvec{u}(t) =-\sum \limits _{j=1}^{r}h_j(\sigma (t))F_{j}\varvec{x}(t).\end{aligned}$$

#### Theorem 1

*The stability PDC control design, as defined in Eq. (*11*), is achieved by finding a positive definite matrix X and matrices* $F_j=M_jX^{-1}$* that satisfies the specified condition.*

$$\begin{aligned} {\left\{ \begin{array}{ll} XA_{i}^T - M_{i}^TB_{i}^T + A_{i}X - B_{i}M_{i}< 0\\ XA_{i}^T + XA_{j}^T - M_{j}^TB_{i}^T - M_{i}^TB_{j}^T + A_{i}X + A_{j}X -B_{i}M_{j} - B_{j}M_{i}< 0, \ i < j\ s.t\ h_{i} \cap h_{j} \ne \varnothing . \\ \end{array}\right. } \end{aligned}$$

#### *Proof*

The Lyapunov function selected is described by Eq. (9). Next, let’s examine the Lyapunov condition:

12 $$\begin{aligned} &\dot{V}(\varvec{x}(t)) = \dot{\varvec{x}}^T(t)P\varvec{x}(t) + \varvec{x}^T(t)P\dot{\varvec{x}}(t)< 0\\&\Leftrightarrow \sum _{i=1}^{r}\sum _{j=1}^{r}h_{i}(\sigma (t))h_{j}(\sigma (t))\varvec{x}^T(t)\{A_{i}^TP - F_{j}^{T}B_{i}^TP + PA_{i} - PB_{i}F_{j}\}\varvec{x}(t)< 0\\&\Leftrightarrow \sum _{i=1}^{r}h_{i}^{2}(\sigma (t))\varvec{x}^T(t)\{A_{i}^TP - F_{i}^TB_{i}^TP + PA_{i} - PB_{i}F_{i}\}\varvec{x}(t)\\&+ \sum _{i=1}^{r}\sum _{i<j}^{r}h_{i}(\sigma (t))h_{j}(\sigma (t))\varvec{x}^T(t)\{A_{i}^TP + A_{j}^TP - F_{j}^TB_{i}^TP - F_{i}^TB_{j}^TP + PA_{i} + PA_{j} -PB_{i}F_{j} - PB_{j}F_{i}\}\varvec{x}(t) < 0. \end{aligned}$$

Equation (12) holds true if and only if:

$$\begin{aligned}{}&{\left\{ \begin{array}{ll} \sum \limits _{i=1}^{r}h_{i}^{2}(\sigma (t))\varvec{x}^T(t)\{A_{i}^TP - F_{i}^TB_{i}^TP + PA_{i} - PB_{i}F_{i}\}\varvec{x}(t)< 0\\ \sum \limits _{i=1}^{r}\sum \limits _{i<j}^{r}h_{i}(\sigma (t))h_{j}(\sigma (t))\varvec{x}^T(t)\{A_{i}^TP + A_{j}^TP - F_{j}^TB_{i}^TP - F_{i}^TB_{j}^TP + PA_{i} + PA_{j} -PB_{i}F_{j} - PB_{j}F_{i}\}\varvec{x}(t)< 0 \end{array}\right. }\\&\Leftrightarrow {\left\{ \begin{array}{ll} A_{i}^TP - F_{i}^TB_{i}^TP + PA_{i} - PB_{i}F_{i}< 0\\ A_{i}^TP + A_{j}^TP - F_{j}^TB_{i}^TP - F_{i}^TB_{j}^TP + PA_{i} + PA_{j} -PB_{i}F_{j} - PB_{j}F_{i} < 0. \\ \end{array}\right. } \end{aligned}$$

Denote $X = P^{-1}$ and multiplying both sides of the inequality by *X* results in:

$$\begin{aligned} {\left\{ \begin{array}{ll} XA_{i}^T - XF_{i}^TB_{i}^T + A_{i}X - B_{i}F_{i}X< 0\\ XA_{i}^T + XA_{j}^T - XF_{j}^TB_{i}^T - XF_{i}^TB_{j}^T + A_{i}X + A_{j}X -B_{i}F_{j}X - B_{j}F_{i}X < 0. \\ \end{array}\right. } \end{aligned}$$

Denote $M_{i} = F_{i}X$, yields the following equation:

$$\begin{aligned} {\left\{ \begin{array}{ll} XA_{i}^T - M_{i}^TB_{i}^T + A_{i}X - B_{i}M_{i}< 0\\ XA_{i}^T + XA_{j}^T - M_{j}^TB_{i}^T - M_{i}^TB_{j}^T + A_{i}X + A_{j}X -B_{i}M_{j} - B_{j}M_{i}< 0, \ i < j\ s.t\ h_{i} \cap h_{j} \ne \varnothing . \\ \end{array}\right. } \end{aligned}$$

This conclude the proof. $\square$

### Robust disturbance rejection

In the context of disturbance rejection in T–S systems, one prominent performance criterion is $H \infty$ performance. This criterion aims to design a controller that minimizes the effect of external disturbances on the system while achieving desired closed-loop stability and performance specifications. The T–S model, incorporating the presence of disturbances, can be represented as follows:

13 $$\begin{aligned} {\left\{ \begin{array}{ll} \varvec{\dot{x}}(t) =h_{i}(\sigma (t))\{A_{i}\varvec{x}(t) + B_{i}\varvec{u}(t) + E_{i}\varvec{v}(t)\}\\ \varvec{y}(t) = h_{i}(\sigma (t))C_{i}\varvec{x}(t) \end{array}\right. } \end{aligned}$$

with $\varvec{v}(t)$ is the disturbance. The performance criterion for disturbance rejection in T–S systems can be expressed by minimizing the value of $\gamma$ to achieve the following property:

14 $$\begin{aligned} \sup \dfrac{\Vert \varvec{y}(t) \Vert _{2} }{\Vert \varvec{v}(t) \Vert _{2}} \le \gamma . \end{aligned}$$

#### Theorem 2

*The feedback gains* $F_{i}$*, responsible for stabilizing the T–S fuzzy model under the presence of disturbances and minimizing the expression* $\gamma$* as shown in Eq. (*14*), can be determined by solving an LMI-based problem where* $M_{i} = F_{i}X$.

$$\begin{aligned} \begin{bmatrix} \begin{pmatrix} -\dfrac{1}{2}\{XA_{i}^T - M_{j}^TB_{i}^T +A_{i}X - B_{i}M_{j}\\ + XA_{j}^T - M_{i}^TB_{j}^T +A_{j}X - B_{j}M_{i}\}\end{pmatrix}&{}-\dfrac{1}{2}(E_{i}+E_{j})&{}\dfrac{1}{2}X(C_{i}+C_{j})^{T}\\ -\dfrac{1}{2}(E_{i}+E_{j})^{T}&{} \gamma ^{2}I&{}0\\ \dfrac{1}{2}(C_{i}+C_{j})X&{}0&{}I\\ \end{bmatrix} \ge 0,\hspace{0.3cm} i \le j\ s.t\ h_{i} \cap h_{j} \ne \varnothing . \end{aligned}$$

#### *Proof*

Let’s assume the existence of a quadratic function $V(\varvec{x}(t)) = \varvec{x}^{T}(t)P\varvec{x}(t)$ and *P* > 0, $\gamma$ $\ge$ 0 $\forall t \ge$ 0, the following conditions hold:

15 $$\begin{aligned} \dot{V}(\varvec{x}(t)) + \varvec{y}^{T}(t)\varvec{y}(t) - \gamma ^{2}\varvec{v}^{T}(t)\varvec{v}(t) \le 0. \end{aligned}$$

Assuming an initial condition $\varvec{x}(0) = 0$, we can derive the following expression:

$$\begin{aligned} V(\varvec{x}(t)) + \int _{0}^{T}(\varvec{y}^{T}(t)\varvec{y}(t) - \gamma ^{2}\varvec{v}^{T}(t)\varvec{v}(t))\le 0. \end{aligned}$$

The inequality $V(\varvec{x}(t)) \ge 0$ implies the following:

$$\begin{aligned} \dfrac{\Vert \varvec{y}(t) \Vert _{2} }{\Vert \varvec{v}(t) \Vert _{2} } \le \gamma . \end{aligned}$$

Taking the derivative of $V(\varvec{x}(t))$, the following expression is obtained:

$$\begin{aligned} \dot{V}(\varvec{x}(t)) =&\ \dot{\varvec{x}}^T(t)P\varvec{x}(t) + \varvec{x}^T(t)P\dot{\varvec{x}}(t)\\ =&\sum _{i=1}^{r}\sum _{j=1}^{r}h_{i}(\sigma (t))h_{j}(\sigma (t))\varvec{x}^T(t)(A_{i} - B_{i}F_{j})^TP\varvec{x}(t) + \sum _{i=1}^{r}\sum _{j=1}^{r}h_{i}(\sigma (t))h_{j}(\sigma (t))\varvec{x}^T(t)P(A_{i} - B_{i}F_{j})\varvec{x}(t) \\&+\sum _{i=1}^{r}h_{i}(\sigma (t))\varvec{v}^T(t)E_{i}^TP\varvec{x}(t) + \sum _{i=1}^{r}h_{i}(\sigma (t))\varvec{x}^T(t)PE_{i}\varvec{v}(t). \end{aligned}$$

Additionally, it follows:

$$\begin{aligned} \varvec{y}^T(t)\varvec{y}(t) - \gamma ^2\varvec{v}^T(t)\varvec{v}(t) = \sum _{i=1}^{r}\sum _{j=1}^{r}h_{i}(\sigma (t))h_{j}(\sigma (t))\bigg \{\varvec{x}^T(t)C_{i}^TC_{j}\varvec{x}(t)-\gamma ^2\varvec{v}^T(t)\varvec{v}(t)\bigg \}. \end{aligned}$$

Therefore, inequality (15) can be expressed as:

16 $$\begin{aligned} \dot{V}(\varvec{x}(t)) + \varvec{y}^T(t)\varvec{y}(t) - \gamma ^2\varvec{v}^T(t)\varvec{v}(t) = \sum _{i=1}^{r}\sum _{j=1}^{r}h_{i}(\sigma (t))h_{j}(\sigma (t))[\varvec{x}^T(t)\ \varvec{v}^T(t)]\times \begin{bmatrix} \begin{pmatrix} (A_{i}-B_{i}F_{j})^TP+P(A_{i}-B_{i}F_{j})\\ +C_{i}^TC_{j}\end{pmatrix} &{}PE_{i} \\ E_{i}^TP &{} -\gamma ^2I \end{bmatrix}\begin{bmatrix} \varvec{x}(t) \\ \varvec{v}(t) \end{bmatrix}\le 0. \end{aligned}$$

Referring to Eq. (16), it can be observed that:

$$\begin{aligned}{} & {} \begin{bmatrix} \begin{pmatrix} -\sum \limits _{i=1}^{r}\sum \limits _{j=1}^{r}h_{i}(\sigma (t))h_{j}(\sigma (t))\{(A_{i}-B_{i}F_{j})^TP \\ +P(A_{i} - B_{i}F_{j})+C_{i}^TC_{j}\} \end{pmatrix} &{} -P\sum \limits _{i=1}^{r}h_{i}(\sigma (t))E_{i} \\ -\sum \limits _{i=1}^{r}h_{i}(\sigma (t))E_{i}^TP &{} \gamma ^2I \end{bmatrix} \ge 0 \\{} & {} \quad \Leftrightarrow \begin{bmatrix} \begin{pmatrix} -\sum \limits _{i=1}^{r}\sum \limits _{j=1}^{r}h_{i}(\sigma (t))h_{j}(\sigma (t))\{(A_{i}-B_{i}F_{j})^TP \\ +P(A_{i} - B_{i}F_{j})\} \end{pmatrix} &{} -P\sum \limits _{i=1}^{r}h_{i}(\sigma (t))E_{i} \\ -\sum \limits _{i=1}^{r}h_{i}(\sigma (t))E_{i}^TP &{} \gamma ^2I \end{bmatrix} -\begin{bmatrix} \sum \limits _{i=1}^{r}\sum \limits _{j=1}^{r}h_{i}(\sigma (t))h_{j}(\sigma (t))C_{i}^TC_{j} &{} 0 \\ 0 &{} 0 \end{bmatrix}\ge 0 \\{} & {} \quad \Leftrightarrow \begin{bmatrix} \begin{pmatrix} -\sum \limits _{i=1}^{r}\sum \limits _{j=1}^{r}h_{i}(\sigma (t))h_{j}(\sigma (t))\{(A_{i}-B_{i}F_{j})^TP \\ +P(A_{i} - B_{i}F_{j})\} \end{pmatrix} &{} -P\sum \limits _{i=1}^{r}h_{i}(\sigma (t))E_{i} \\ -\sum \limits _{i=1}^{r}h_{i}(\sigma (t))E_{i}^TP &{} \gamma ^2I \end{bmatrix} - \begin{bmatrix} \sum \limits _{i=1}^{r}h_{i}(\sigma (t))C_{i}^T \\ 0 \end{bmatrix}\begin{bmatrix} \sum \limits _{i=1}^{r}h_{i}(\sigma (t))C_{i}&0 \end{bmatrix} \ge 0. \end{aligned}$$

Applying the Schur complement, the result can be obtained as follows:

$$\begin{aligned}{} & {} \begin{bmatrix} \begin{pmatrix} -\sum \limits _{i=1}^{r}\sum \limits _{j=1}^{r}h_{i}(\sigma (t))h_{j}(\sigma (t))\{(A_{i}-B_{i}F_{j})^TP \\ +P(A_{i} - B_{i}F_{j})\} \end{pmatrix} &{} -P\sum \limits _{i=1}^{r}h_{i}(\sigma (t))E_{i} &{} \sum \limits _{i=1}^{r}h_{i}(\sigma (t))C_{i}^T \\ -\sum \limits _{i=1}^{r}h_{i}(\sigma (t))E_{i}^TP &{} \gamma ^2I &{} 0 \\ \sum \limits _{i=1}^{r}h_{i}(\sigma (t))C_{i}&{} 0 &{} I \end{bmatrix} \ge 0 \\{} & {} \quad \Leftrightarrow \sum \limits _{i=1}^{r}\sum \limits _{j=1}^{r}h_{i}(\sigma (t))h_{j}(\sigma (t)) \begin{bmatrix} \begin{pmatrix} -\frac{1}{2}\{(A_{i}-B_{i}F_{j})^TP +P(A_{i} - B_{i}F_{j})\\ +(A_{j}-B_{j}F_{i})^TP +P(A_{j} - B_{j}F_{i})\} \end{pmatrix} &{} -\frac{1}{2}P(E_{i} + E_{j}) &{} \frac{1}{2}(C_{i} + C_{j})^T \\ -\frac{1}{2}(E_{i} + E_{j})^TP &{} \gamma ^2I &{} 0 \\ \frac{1}{2}(C_{i} + C_{j})&{} 0 &{} I \end{bmatrix} \ge 0. \end{aligned}$$

The result can be deduced:

$$\begin{aligned} \begin{bmatrix} \begin{pmatrix} -\frac{1}{2}\{(A_{i}-B_{i}F_{j})^TP +P(A_{i} - B_{i}F_{j})\\ +(A_{j}-B_{j}F_{i})^TP +P(A_{j} - B_{j}F_{i})\} \end{pmatrix} &{} -\frac{1}{2}P(E_{i} + E_{j}) &{} \frac{1}{2}(C_{i} + C_{j})^T \\ -\frac{1}{2}(E_{i} + E_{j})^TP &{} \gamma ^2I &{} 0 \\ \frac{1}{2}(C_{i} + C_{j})&{} 0 &{} I \end{bmatrix} \ge 0. \end{aligned}$$

This concludes the proof. $\square$

### Input constraints

In addition to stability and disturbance rejection, addressing input constraints is crucial in ensuring safe and reliable operation of the system. This section focuses on the consideration and incorporation of input constraints into the control design framework for the T–S fuzzy model.

#### Theorem 3

*Assuming the initial condition* $\varvec{x}(0)$* is known, the constraint* $\Vert \varvec{u}(t) \Vert _2 \le \mu$* can be maintained at all times* $t \ge 0$* by satisfying the following LMI conditions:*

$$\begin{aligned} \begin{bmatrix} \tau &{}\varvec{x}^{T}(0)\\ \varvec{x}(0)&{}X \end{bmatrix} \ge 0, \hspace{1cm} \begin{bmatrix} \dfrac{X}{\tau }&{}M_{i}^{T}\\ M_{i}&{}\mu ^{2}I \end{bmatrix} \ge 0 \end{aligned}$$

*where* $$X = P^{-1},$$ $$M_{i} = F_{i}X,$$ $$\tau > 0$$ *and* $$\mu > 0$$.

#### *Proof*

Let us assume the Lyapunov function such as:

$$\begin{aligned} V(\varvec{x}(t)) = \varvec{x}^{T}(t)P\varvec{x}(t) \end{aligned}$$

and

$$\begin{aligned} \varvec{x}^{T}(0)P\varvec{x}(0) \le \tau \ \Leftrightarrow \ 1 - \dfrac{\varvec{x}^{T}(0)P\varvec{x}(0)}{\tau } \ge 0. \end{aligned}$$

Applying the Schur complement yields:

17 $$\begin{aligned} \begin{bmatrix} \tau &{}\ \varvec{x}^{T}(0)\\ \varvec{x}(0)&{}\ P^{-1} \end{bmatrix} \ge 0 \end{aligned}$$

with

$$\begin{aligned} {\Vert \varvec{u}(t) \Vert }_2 \le \mu \ {}&\Leftrightarrow \ \varvec{u}^{T}(t)\varvec{u}(t) \le \mu ^{2} \ \Leftrightarrow \ \varvec{u}^{T}(t)\varvec{u}(t) = \sum _{i=1}^{r}\sum _{j=1}^{r}h_{i}(\sigma (t))h_{j}(\sigma (t))\varvec{x}^{T}(t)F_{i}^TF_{j}\varvec{x}(t) \le \mu ^{2} \\&\Leftrightarrow \dfrac{1}{\mu ^{2}}\sum _{i=1}^{r}\sum _{j=1}^{r}h_{i}(\sigma (t))h_{j}(\sigma (t))\varvec{x}^{T}(t)F_{i}^TF_{j}\varvec{x}(t) \le 1. \end{aligned}$$

With $\forall$ $t \ge 0$ and $\dfrac{\varvec{x}^{T}(t)X^{-1}\varvec{x}(t)}{\tau } \le \dfrac{\varvec{x}^{T}(0)X^{-1}\varvec{x}(0)}{\tau } \le 1$, we can conclude if

$$\begin{aligned} \dfrac{1}{\mu ^{2}}\sum _{i=1}^{r}\sum _{j=1}^{r}h_{i}(\sigma (t))h_{j}(\sigma (t))\varvec{x}^{T}(t)F_{i}^TF_{j}\varvec{x}(t) \le \dfrac{\varvec{x}^{T}(t)X^{-1}\varvec{x}(t)}{\tau } \end{aligned}$$

then

$$\begin{aligned}{}&\sum _{i=1}^{r}\sum _{j=1}^{r}h_{i}(\sigma (t))h_{j}(\sigma (t))\varvec{x}^{T}(t)\bigg \{\dfrac{1}{\mu ^{2}}F_{i}^TF_{j} - \dfrac{X^{-1}}{\tau }\bigg \}\varvec{x}(t) \le 0 \\&\Leftrightarrow \dfrac{1}{2}\sum _{i=1}^{r}\sum _{j=1}^{r}h_{i}(\sigma (t))h_{j}(\sigma (t))\varvec{x}^{T}(t)\bigg \{\dfrac{1}{\mu ^{2}}F_{i}^TF_{j} + \dfrac{1}{\mu ^{2}}F_{j}^TF_{i} - \dfrac{2X^{-1}}{\tau }\bigg \}\varvec{x}(t) \le 0 \\&\Leftrightarrow \dfrac{1}{2}\sum _{i=1}^{r}\sum _{j=1}^{r}h_{i}(\sigma (t))h_{j}(\sigma (t))\varvec{x}^{T}(t)\bigg \{\dfrac{1}{\mu ^{2}}F_{i}^TF_{i} + \dfrac{1}{\mu ^{2}}F_{j}^TF_{j} - \dfrac{1}{\mu ^2}(F_{i}^{T} - F_{j}^{T})(F_{i} - F_{j}) - \dfrac{2X^{-1}}{\tau }\bigg \}\varvec{x}(t) \le 0. \end{aligned}$$

Consider:

$$\begin{aligned}{}&\dfrac{1}{2}\sum _{i=1}^{r}\sum _{j=1}^{r}h_{i}(\sigma (t))h_{j}(\sigma (t))\varvec{x}^{T}(t)\bigg \{\dfrac{1}{\mu ^{2}}F_{i}^TF_{i} + \dfrac{1}{\mu ^{2}}F_{j}^TF_{j} - \dfrac{2X^{-1}}{\tau }\bigg \}\varvec{x}(t) \le 0 \\&\Leftrightarrow \sum _{i=1}^{r}h_{i}(\sigma (t))\varvec{x}^{T}(t)\bigg \{\dfrac{1}{\mu ^{2}}F_{i}^TF_{i} - \dfrac{X^{-1}}{\tau }\bigg \}\varvec{x}(t) \le 0. \end{aligned}$$

Therefore,

$$\begin{aligned} \dfrac{1}{\mu ^{2}}F_{i}^TF_{i} - \dfrac{X^{-1}}{\tau } \le 0 \ \Leftrightarrow \ \dfrac{1}{\mu ^{2}}XF_{i}^TF_{i}X - \dfrac{X}{\tau } \le 0. \end{aligned}$$

Applying the Schur complement yields:

$$\begin{aligned} \begin{bmatrix} \dfrac{X}{\tau }&{}\ M_{i}^T\\ M_{i}&{}\ \mu ^2I \end{bmatrix} \ge 0. \end{aligned}$$

This conclude the proof. $\square$

### Output constraints

The section on output constraints delves into the restrictions placed on the system’s output variables, which are crucial for preserving system stability and achieving desired performance. To handle these constraints within the framework of the Takagi–Sugeno fuzzy model, a theorem similar to the one presented for input constraints will be introduced.

#### Theorem 4

*Suppose that the initial condition* $\varvec{x}(0)$* is given, and the constraint* $\parallel \varvec{y}(t) \parallel _2 \le \lambda$* is satisfied for all* $t \ge 0$* if the linear matrix inequality (LMI) is met.*

$$\begin{aligned} \begin{bmatrix} \tau &{}\varvec{x}^{T}(0)\\ \varvec{x}(0)&{}X \end{bmatrix} \ge 0, \hspace{1cm} \begin{bmatrix} \dfrac{X}{\tau }&{}XC_{i}^{T}\\ C_{i}X&{}\lambda ^{2}I \end{bmatrix} \ge 0 \end{aligned}$$

*with* $$X = P^{-1},$$ $$M_{i} = F_{i}X,$$ $$\tau > 0$$ *and*$$\lambda >0.$$

The proof of this theorem will follow the same approach as in the previous section.

In the main results section, it is important to emphasize the capability of LMI to combine multiple control theorems, such as disturbance rejection and input-output constraints, into a unified framework. By simultaneously solving the corresponding LMI conditions, the control strategy can effectively address both disturbance rejection and input-output constraints. This approach allows for the synthesis of controllers that provide robust disturbance rejection while ensuring that the system operates within specified input-output limits. The use of LMIs enables the integration of these control objectives and constraints, simplifying the design process and facilitating the development of a comprehensive and efficient control strategy. By leveraging the power of LMIs, it becomes possible to achieve desired performance and robustness requirements while satisfying multiple control objectives simultaneously.

#### *Remark 1*

To assess the effectiveness of the proposed control strategies, simulations were conducted for each of the four cases using the framework of LMI. In the first case, the focus was on stabilization (Theorem 1), where the LMI conditions were employed to ensure the stability of the system. For the second case, the objective was disturbance rejection (Theorem 2), and LMIs were utilized to design controllers that effectively attenuate disturbances. In the third case, both disturbance rejection and output constraint were considered, combining Theorem 2 and Theorem 4. The LMIs played a crucial role in achieving disturbance rejection while satisfying the specified output constraint. Finally, in the fourth case, the aim was to address disturbance rejection along with input-output constraints, which involved the integration of Theorem 2, Theorem 3, and Theorem 4. Through the application of LMIs, controllers were designed to mitigate disturbances while ensuring the system operated within the prescribed input-output limits. These simulations, conducted with the aid of LMIs and LMI theorems, demonstrate the efficacy and versatility of the proposed control strategies in addressing various control objectives and constraints.

- First case: Stabilization (Theorem 1).
- Second case: Disturbance Rejection (Theorem 2).
- Third case: Disturbance Rejection with Output Constraint (Theorem 2 + Theorem 4).
- Fourth case: Disturbance Rejection with Input-Output Constraints (Theorem 2 + Theorem 3 + Theorem 4).

## Results

This section presents the findings and outcomes obtained from the application of the stability theorem, disturbance rejection theorem, constraint output theorem, and constraint input theorem discussed in the previous section. These results showcase the efficiency and suitability of the suggested methodologies in achieving system stability, robust disturbance rejection, and satisfying input and output constraints within the T–S system. The analysis and evaluation of the results provide valuable insights into the performance and control capabilities of the developed control strategies. Considering the model parameters specified in the Table 1, the following initial conditions have been selected:

$$\begin{aligned} \phi \in [\dfrac{\pi }{6};\dfrac{-\pi }{6}]\ (rad),\ \theta \in [0;2\pi ]\ (rad),\ \dot{\phi } \in [1.5;2]\ (rad/s),\ \dot{\theta } \in [1;1.5]\ (rad/s). \end{aligned}$$

By applying the parameters, one can determine the maximum and minimum values of premise variables, see Table 2.

**Table 2:** Premise variable bounds.

Premise variable	Max	Min
$\sigma _1(t)$	$1.058\times 10^{4}$	$8.198\times 10^{3}$
$\sigma _2(t)$	$7.029\times 10^{-3}$	$6.821\times 10^{-3}$
$\sigma _3(t)$	$-2.867\times 10^{-4}$	$-3.931\times 10^{-4}$
$\sigma _4(t)$	$-1.500\times 10^{-3}$	$-1.732\times 10^{-3}$
$\sigma _5(t)$	$1.042\times 10^{-3}$	$6.993\times 10^{-4}$
$\sigma _6(t)$	$-2.294\times 10^{-3}$	$-2.774\times 10^{-3}$
$\sigma _7(t)$	$1.743\times 10^{-4}$	$4.796\times 10^{-5}$
$\sigma _8(t)$	$-9.101\times 10^{-4}$	$-1.134\times 10^{-3}$

The membership functions are represented as follows:

$$\begin{aligned}{}&w_{10} = \dfrac{\sigma _{1max} - \sigma _{1}(t)}{\sigma _{1max} - \sigma _{1min}},\ \ w_{11} = 1 - w_{10},\ \ w_{20} = \dfrac{\sigma _{2max} - \sigma _{2}(t)}{\sigma _{2max} - \sigma _{2min}},\ \ w_{21} = 1 - w_{20},\\&w_{30} = \dfrac{\sigma _{3max} - \sigma _{3}(t)}{\sigma _{3max} - \sigma _{3min}},\ \ w_{31} = 1 - w_{30},\ \ w_{40} = \dfrac{\sigma _{4max} - \sigma _{4}(t)}{\sigma _{4max} - \sigma _{4min}},\ \ w_{41} = 1 - w_{40},\\&w_{50} = \dfrac{\sigma _{5max} - \sigma _{5}(t)}{\sigma _{5max} - \sigma _{5min}},\ \ w_{51} = 1 - w_{50},\ \ w_{60} = \dfrac{\sigma _{6max} - \sigma _{6}(t)}{\sigma _{6max} - \sigma _{6min}},\ \ w_{61} = 1 - w_{60},\\&w_{70} = \dfrac{\sigma _{7max} - \sigma _{7}(t)}{\sigma _{7max} - \sigma _{7min}},\ \ w_{71} = 1 - w_{70},\ \ w_{80} = \dfrac{\sigma _{8max} - \sigma _{8}(t)}{\sigma _{8max} - \sigma _{8min}},\ \ w_{81} = 1 - w_{80}. \end{aligned}$$

To avoid excessive length in this article, the state matrix *A*, control matrix *B*, and common positive definite matrix *P* will be represented using a general formula, as follows:

$$\begin{aligned}{} & {} A_{\zeta } = \begin{bmatrix} 0&{} 0&{} 1&{} 0\\ 0&{} 0&{} 0&{} 1\\ \sigma _{1q_1}\sigma _{2q_2}&{} 0&{} \sigma _{1q_1}\sigma _{3q_3}&{} \sigma _{1q_1}\sigma _{5q_5}\\ \sigma _{1q_1}\sigma _{6q_6}&{} 0&{} \sigma _{1q_1}\sigma _{7q_7}&{} \sigma _{1q_1}\sigma _{8q_8} \end{bmatrix}, \\{} & {} B_{\zeta } = \begin{bmatrix} 0\\ 0\\ \sigma _{1q_1}\sigma _{4q_4}\\ 4s_{1}s_7\sigma _{1q_1} \end{bmatrix} \end{aligned}$$

where

$$\begin{aligned} \zeta =\ q_8 + 2(q_7-1) + 4(q_6-1) + 8(q_5-1) + 16(q_4-1) + 32(q_3-1) + 64(q_2-1) + 128(q_1-1) \end{aligned}$$

and $q_1$, $q_2$, ... $q_8 \in [1,2]$. The value of the premise variable can be determined as:

$$\begin{aligned}{}&\sigma _{11} = \sigma _{1max},\ \sigma _{12} = \sigma _{1min},\ \sigma _{21} = \sigma _{2max},\ \sigma _{22} = \sigma _{2min},\\&\sigma _{31} = \sigma _{3max},\ \sigma _{32} = \sigma _{3min},\ \sigma _{41} = \sigma _{4max},\ \sigma _{42} = \sigma _{4min},\\&\sigma _{51} = \sigma _{5max},\ \sigma _{52} = \sigma _{5min},\ \sigma _{61} = \sigma _{6max},\ \sigma _{62} = \sigma _{6min},\\&\sigma _{71} = \sigma _{7max},\ \sigma _{72} = \sigma _{7min},\ \sigma _{81} = \sigma _{8max},\ \sigma _{82} = \sigma _{8min}. \end{aligned}$$

In the study, four distinct cases were investigated. The first case focused on achieving stability in the T–S fuzzy model by computing the matrix *P* based on the stability condition. In the second case, the objective was to attenuate and compensate for disturbances by optimizing the $\gamma$. The third case addressed both disturbance rejection and output constraints, ensuring effective rejection of disturbances while operating within $\phi$ angle limit. Lastly, the fourth case expanded the analysis to include input voltage *u*, providing a comprehensive control strategy for effective disturbance rejection while satisfying both input and output limitations. The T–S fuzzy model was implemented, and the corresponding control design based on the LMI theorems was applied. The simulation outcomes illustrate the successful achievement of the desired objectives in each case, showcasing the stability, disturbance rejection capability, and adherence to input and output constraints. The following sections present the specific simulation results and performance evaluations for each case.

The positive definite matrix *P* results are:

- First case : $$P = \begin{bmatrix} 5273.856&{} 7.819&{} 1589.074&{} 352.460\\ 7.819&{} 0.036&{} 2.431&{} 0.600\\ 1589.074&{} 2.431&{} 491.487&{} 117.597\\ 352.460&{} 0.600&{} 117.597&{} 35.810 \end{bmatrix}$$.
- Second case: $$P = \begin{bmatrix} 612.624&{} 0.136&{} 124.658&{} 1.930\\ 0.136&{} 0.003&{} 0.030&{} 0.004\\ 124.658&{} 0.030&{} 27.732&{} 1.952\\ 1.930&{} 0.004&{} 1.952&{} 2.467 \end{bmatrix}$$.
- Third case: $$P = \begin{bmatrix} 219567.133&{} 7.252&{} 2064.767&{} 182.836\\ 7.252&{} 0.001&{} 0.069&{} 0.007\\ 2064.767&{} 0.069&{} 26.294&{} 4.307\\ 182.836&{} 0.007&{} 4.307&{} 2.702 \end{bmatrix}$$.
- Fourth case: $$P = \begin{bmatrix} 121403.315 &{} 4.2095 &{} 829.5755 &{} 10.046 \\ 4.2095 &{} 0.0005 &{} 0.029 &{} 0.0005 \\ 829.5755 &{} 0.029 &{} 10.0005 &{} 0.8125 \\ 10.046 &{} 0.0005 &{} 0.8125 &{} 0.8295 \end{bmatrix}$$.

Here are the matrices *A* and *B* for the selected rules ($1{st}$, $16{th}$, $64{th}$, and $256{th}$), which are similar across the four cases:

- $$1{st}$$ Rule: $$A_{1} = \begin{bmatrix} 0&{} 0&{} 1&{} 0\\ 0&{} 0&{} 0&{} 1\\ 74.394&{} 0&{} -3.035&{} 1.844\\ -24.278&{} 0&{} 11.025&{} -9.632 \end{bmatrix}$$, $$B_{1} = \begin{bmatrix} 0\\ 0\\ -15.873\\ 19.039 \end{bmatrix}$$.
- $$16{th}$$ Rule: $$A_{16} = \begin{bmatrix} 0&{} 0&{} 1&{} 0\\ 0&{} 0&{} 0&{} 1\\ 74.394&{} 0&{} -3.035&{} 0.508\\ -29.357&{} 0&{} 7.402&{} -12.000 \end{bmatrix}$$, $$B_{16} = \begin{bmatrix} 0\\ 0\\ -15.873\\ 19.039 \end{bmatrix}$$.
- $$64{th}$$ Rule: $$A_{64} = \begin{bmatrix} 0&{} 0&{} 1&{} 0\\ 0&{} 0&{} 0&{} 1\\ 74.394&{} 0&{} -4.161&{} 0.508\\ -29.357&{} 0&{} 7.402&{} -12.000 \end{bmatrix}$$, $$B_{64} = \begin{bmatrix} 0\\ 0\\ -18.329\\ 19.039 \end{bmatrix}$$.
- $$256{th}$$ Rule: $$A_{256} = \begin{bmatrix} 0&{} 0&{} 1&{} 0\\ 0&{} 0&{} 0&{} 1\\ 55.915&{} 0&{} -3.223&{} 0.393\\ -22.740&{} 0&{} 5.7332&{} -9.295 \end{bmatrix}$$, $$B_{256} = \begin{bmatrix} 0\\ 0\\ -14.197\\ 14.747 \end{bmatrix}$$.

Here are some selected control gains obtained for each case to demonstrate the differences in the results:

- First case :
  $$F_{1} = \begin{bmatrix} -144.826&-0.210&-42.339&-9.764 \end{bmatrix}$$, $$F_{16} = \begin{bmatrix} -185.081&-0.272&-55.010&-12.945 \end{bmatrix}$$, $$F_{64} = \begin{bmatrix} -240.728&-0.359&-72.559&-17.367 \end{bmatrix}$$, $$F_{256} = \begin{bmatrix} -272.466&-0.407&-82.157&-19.516 \end{bmatrix}$$.
- Second case:
  $$F_{1} = \begin{bmatrix} -27.682&-0.006&-4.761&-0.467 \end{bmatrix}$$, $$F_{16} = \begin{bmatrix} -30.701&-0.006&-5.555&-0.611 \end{bmatrix}$$, $$F_{64} = \begin{bmatrix} -33.2463&-0.007&-6.225&-0.718 \end{bmatrix}$$, $$F_{256} = \begin{bmatrix} -38.650&-0.008&-7.331&-0.727 \end{bmatrix}$$.
- Third case: $$F_{1} = \begin{bmatrix} -3269.346&-0.108&-31.570&-3.676 \end{bmatrix}$$, $$F_{16} = \begin{bmatrix} -3332.061&-0.110&-32.538&-3.905 \end{bmatrix}$$, $$F_{64} = \begin{bmatrix} -3106.949&-0.103&-30.407&-3.692 \end{bmatrix}$$, $$F_{256} = \begin{bmatrix} -3920.514&-0.130&-38.025&-4.341 \end{bmatrix}$$.
- Fourth case:
  $$F_{1} = \begin{bmatrix} -2108.884&-0.073&-15.537&-0.781 \end{bmatrix}$$, $$F_{16} = \begin{bmatrix} -2150.347&-0.075&-16.317&-0.935 \end{bmatrix}$$, $$F_{64} = \begin{bmatrix} -2039.306&-0.071&-15.467&-0.890 \end{bmatrix}$$, $$F_{256} = \begin{bmatrix} -2251.364&-0.078&-16.932&-0.862 \end{bmatrix}$$.

Here are the membership functions for the rules in the T–S fuzzy model:

$$\begin{aligned} h_{1}(\sigma (t))&= w_{10}\times w_{20}\times w_{30}\times w_{40}\times w_{50}\times w_{60} \times w_{70}\times w_{80},\\ h_{16}(\sigma (t))&= w_{10}\times w_{20}\times w_{30}\times w_{41}\times w_{50}\times w_{60} \times w_{70}\times w_{80},\\ h_{64}(\sigma (t))&= w_{10}\times w_{21}\times w_{30}\times w_{40}\times w_{50}\times w_{60} \times w_{70}\times w_{80},\\ h_{256}(\sigma (t))&= w_{11}\times w_{21}\times w_{31}\times w_{41}\times w_{51}\times w_{61} \times w_{71}\times w_{81}. \end{aligned}$$

The previously presented results include the matrix *P* and gain *F* obtained from the LMI solution. In addition, for cases 2, 3, and 4 utilizing the disturbance rejection method, the $\gamma$ value has been optimized. Specifically, the optimal $\gamma$ values found for cases 2, 3, and 4 are 1.41, 1.308, and 6, respectively.

**Figure 2:**
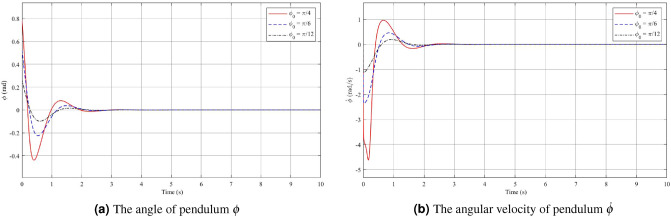
Angle and angular velocity of the pendulum with changing initial conditions in the first case: stabilization theorem.

**Figure 3:**
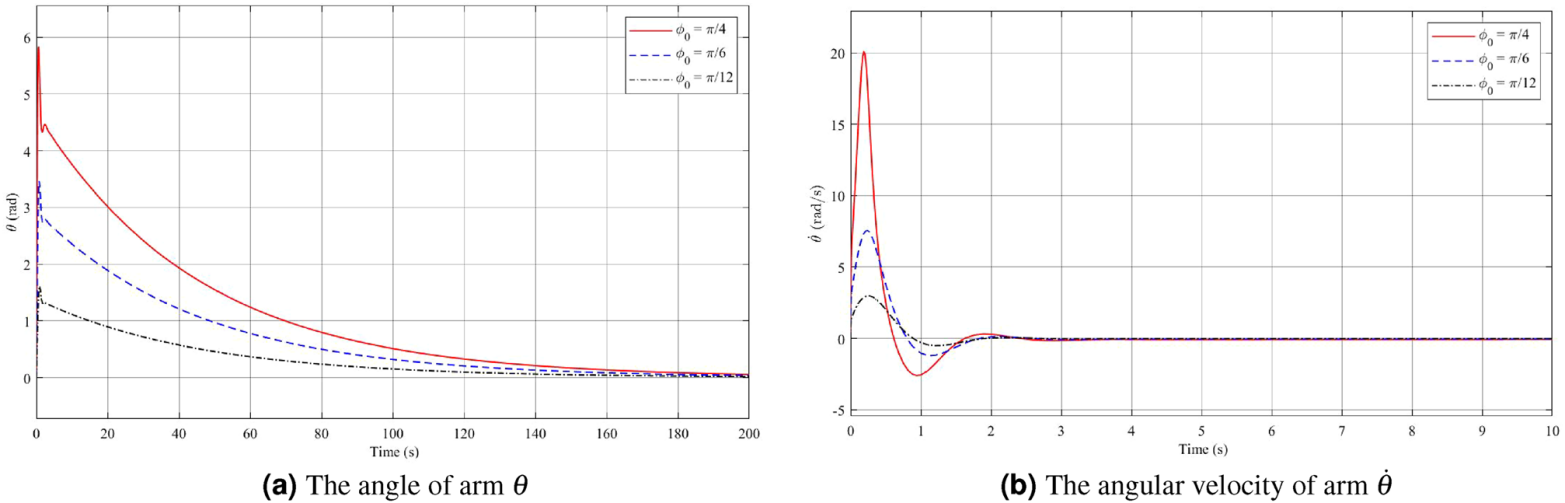
Angle and angular velocity of the arm with changing initial conditions in the first case: stabilization theorem.

**Figure 4:**
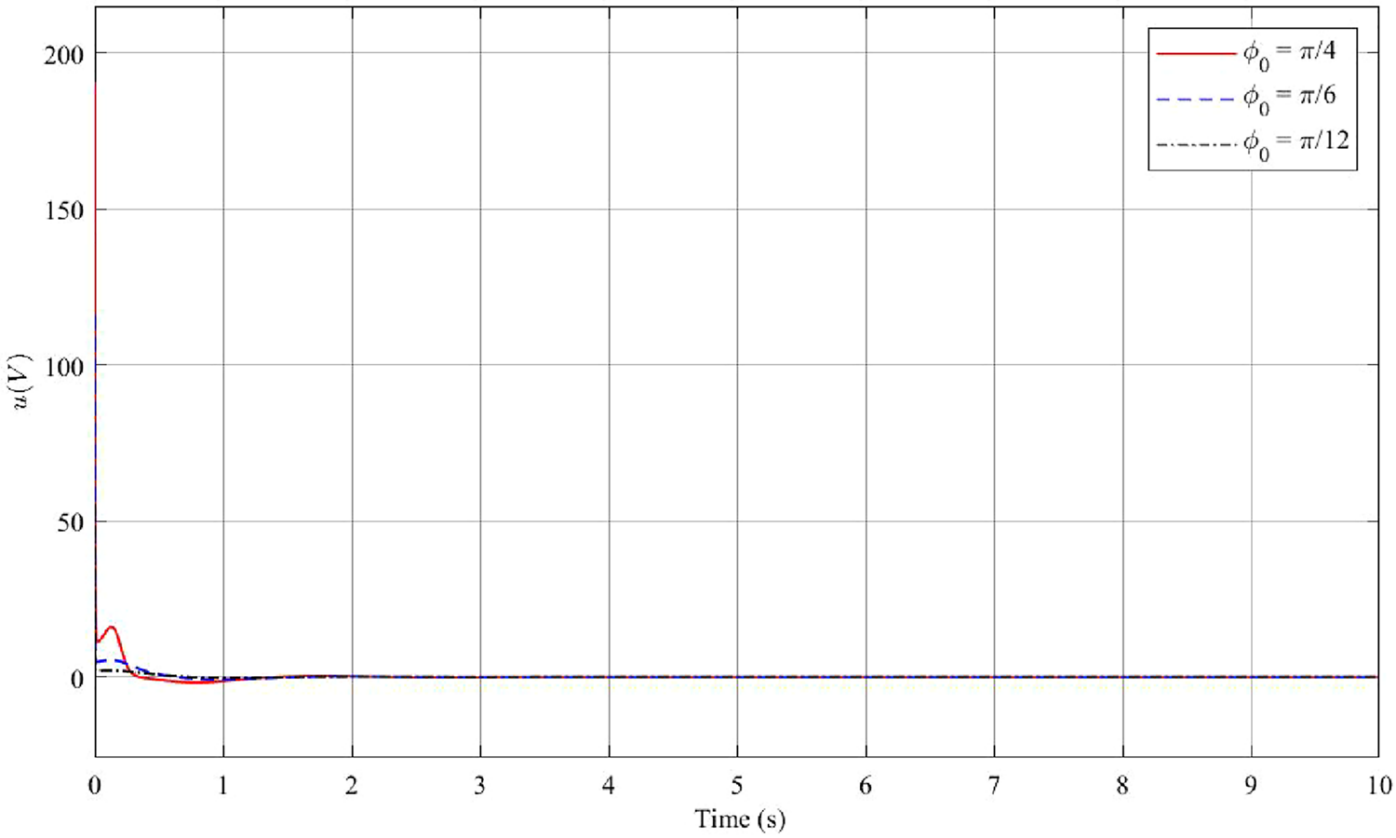
The voltage *u* with changing initial conditions in the first case: stabilization theorem.

During testing and evaluation, remarkable results indicate that the system can effectively control the maximum deflection angle within the [$\dfrac{-56\pi }{180}; \dfrac{56\pi }{180}$] range. However, consider practical limitations of certain mechanisms within the system, the optimal operating range of the controller for the initial deviation angle $\phi _0$ lies within the range of [$-\pi /4; \pi /4$]. In order to investigate the dependence of the fuzzy controller on the system’s initial conditions, small changes have been made to vary the value of the original deflection angle, denoted as $\phi _0$. Simulation results in the first case with different initial conditions are presented, see Figs. 2, 3 and 4. The red line represents the case where $\phi _0$ is set to $\dfrac{\pi }{4}$, the blue line corresponds to $\dfrac{\pi }{6}$, and the black line corresponds to $\dfrac{\pi }{12}$. By analyzing the system’s response under these varying initial conditions, the efficiency and robustness of the stabilization control method can be evaluated and some necessary remarks can be drawn.

In Fig. 2a, the results of the pendulum angle for the First case are presented. The initial angle $\phi _0$ falls within the operational range of the fuzzy controller. As the initial deflection pendulum angle varies, the controller reaches a steady state after approximately 3s. It is observed that larger initial deflection angles lead to longer settling times and increased overshoot. Specifically, when $\phi _0=\dfrac{\pi }{4}$, the settling time is about 3s with an overshoot of 0.439 rad. On the other hand, for the cases where the initial deviation angles are $\phi _0=\dfrac{\pi }{6}$ and $\phi _0=\dfrac{\pi }{12}$, the system achieves steady-state in approximately 2.5s and 2.3s, respectively, with overshoot values of 0.2251 rad and 0.096 rad. Figure 2b displays the angular velocity of the pendulum. As the pendulum angle increases, the magnitude of the voltage required to drive the motor shaft also increases. This leads to larger variations in the pendulum’s angular velocity. The figure illustrates the maximum pendulum angular velocity in the cases of $\phi _0=\dfrac{\pi }{4}$, $\dfrac{\pi }{6}$ and $\dfrac{\pi }{12}$, which are approximately 4.957 rad/s, 2.346 rad/s, and 1.106 rad/s, respectively. It is evident that during the initial period of motion, the velocities of the system undergo rapid changes in a short time interval. Specifically, for $\phi _0=\dfrac{\pi }{4}$, the pendulum angular velocity varies by approximately 4 rad/s, for $\phi _0=\dfrac{\pi }{6}$, it varies by 2 rad/s, and for $\phi _0=\dfrac{\pi }{12}$, it varies by 1 rad/s. Similarly to Fig. 2a,b, the arm angle and angular velocity exhibit a proportional relationship with the magnitude of the initial deflection $\phi _0$. When observing Fig. 3a,b, it is apparent that the variation in these two values is greater compared to the pendulum angle and pendulum angular velocity. This phenomenon can be attributed to the fact that in the system, the pendulum arm is the component directly impacted by the motor, while the pendulum undergoes a secondary effect. Once the pendulum angle has converged to its equilibrium position after approximately 3s, the arm angular velocity also tends to stabilize at zero. However, due to the initial significant variation, the arm angle takes a longer time to converge to zero, typically around 200s. Figure 4 demonstrates that as the initial deflection angle increases, the required starting voltage for the system also increases. Specifically, for initial deviation angle values of $\phi _0=\dfrac{\pi }{4}$, $\phi _0=\dfrac{\pi }{6}$, and $\phi _0=\dfrac{\pi }{12}$, the corresponding starting voltage values are 189.2 V, 115.5 V, and 55.2 V, respectively. Despite the variations in the initial angles, Theorem 1 guarantees the stability of both the pendulum angle and the arm angle. This highlights the robustness of the control strategy in maintaining stability across different initial conditions.

**Figure 5:**
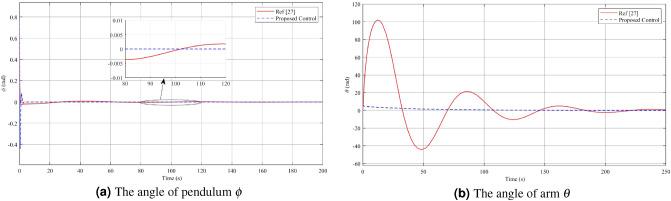
Comparison of pendulum and arm angles: Ref^27^ vs. proposed control.

The response of the pendulum angle $\phi$ and arm angle $\theta$ is compared between the T–S 256 fuzzy rules (Proposed Control) and the T–S 8 fuzzy rules^27^. In the study employing 8 fuzzy rules, the authors applied the T–S fuzzy descriptor model to control the RIP using the identical parameters as presented in Table 1. The Fig. 5 provides a visual representation of the comparison results for these controllers. Upon considering a condition where the initial deflection angle of the pendulum is $\frac{\pi }{4}$, a comparison between the T–S 8 fuzzy rules^27^ and the proposed controller reveals notable differences. Figure 5a demonstrates the behavior of the angle $\phi$: With the T–S 8 fuzzy rules, the overshoot value is merely 0.02 rad, considerably smaller than the proposed controller’s 0.4395 rad. However, it is noteworthy that despite the minor overshoot, the T–S 8 fuzzy rules controller maintains the angle $\phi$ within a small amplitude fluctuation of 0.004 rad after approximately 200 seconds, eventually reaching a steady state. In contrast, the proposed control controller achieves a steady state in just 4 seconds. Moving on to Fig. 5b, which depicts the angle $\theta$: When utilizing the proposed controller, the overshoot is only 5.863 rad, whereas the T–S 8 fuzzy rules controller results in an overshoot of up to 101.7 rad, approximately 17.5 times larger than that of the proposed control. Additionally, the settling time for the angle $\theta$ with the proposed controller is significantly faster compared to the T–S 8 fuzzy rules controller. The proposed controller reaches a steady state in around 200 seconds, while the T–S 8 fuzzy rules controller takes approximately 250 seconds. In conclusion, the proposed controller exhibits a faster setting time than the T–S 8 fuzzy rules controller. Moreover, although the T–S 8 fuzzy rules controller shows improved performance in terms of the angle $\phi$ overshoot, it leads to a substantially larger overshoot in the angle $\theta$ compared to the proposed controller.

**Figure 6:**
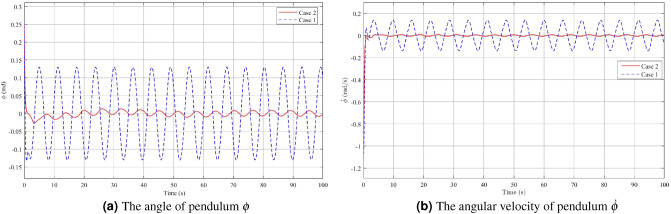
Angle and angular velocity of the pendulum in the first case and second case.

**Figure 7:**
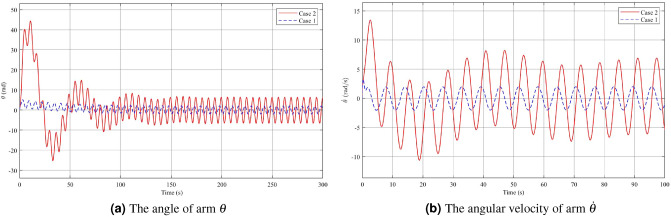
Angle and angular velocity of the arm in the first case and second case.

**Figure 8:**
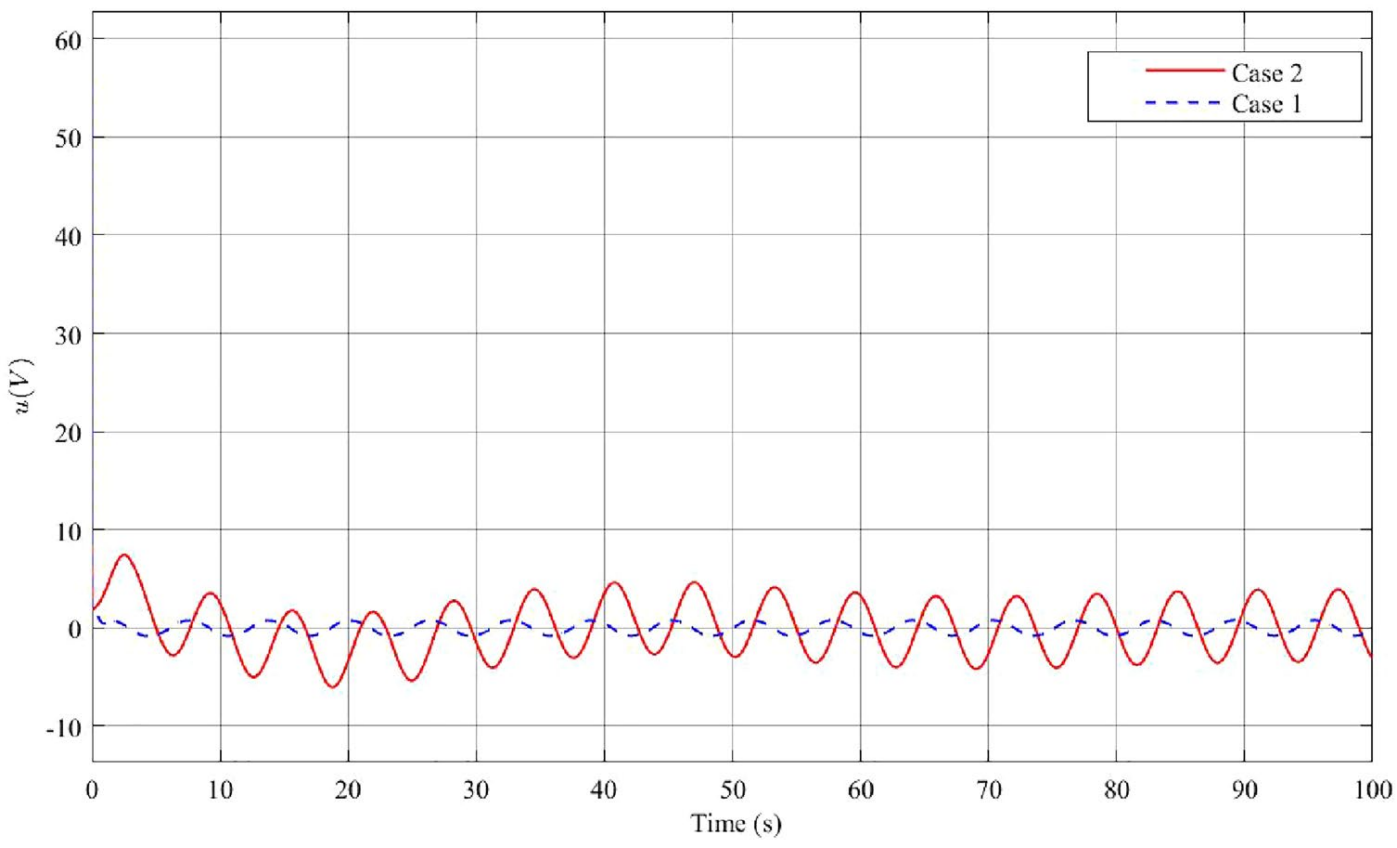
The voltage *u* in the first case and second case.

In the second case, which involves disturbance rejection using Theorem 2, the simulation results are shown in Figs. 6, 7 and 8. The impact of the disturbance $\varvec{v}(t)=3\sin (\pi t)$ on the angle acceleration of the pendulum and the pendulum arm can be observed from the figures, which demonstrate the effectiveness of the controller in mitigating its effects Fig. 6a,b. When a fuzzy controller is employed in the presence of disturbance, the pendulum angle oscillates around the equilibrium position with an amplitude of 0.13 rad and an angular velocity of 0.1395 rad/s. However, when a disturbance rejection controller is used, the swing of the pendulum angle from the equilibrium position is significantly reduced. The amplitude of the pendulum angle oscillation with the controller is only 0.007 rad, and the angular velocity is 0.008 rad/s. These results highlight the superior disturbance rejection capability of the controller, demonstrating its effectiveness in minimizing the impact of disturbance on the system’s behavior. By examining Figs. 7a,b, and 8, it becomes evident that when employing a disturbance rejection controller, the values of arm angle, arm velocity, and control voltage exhibit more pronounced fluctuations compared to using a conventional controller. This behavior is expected since the disturbance rejection controller adjusts the control voltage to counteract the influence of disturbance and maintain the pendulum’s stability at the equilibrium position. As a result, the fluctuations in arm angle and arm angular velocity become more significant. The increased fluctuation in the input and outputs is a trade-off for achieving improved disturbance rejection performance, ensuring the stability of the system in the presence of disturbances.

**Figure 9:**
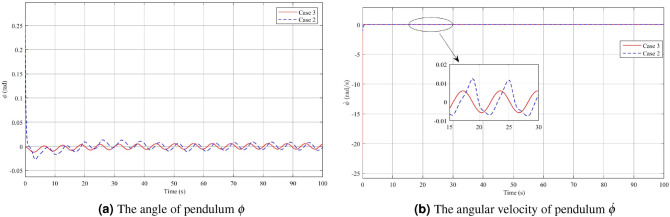
Angle and Angular velocity of the pendulum in the second case and third case.

**Figure 10:**
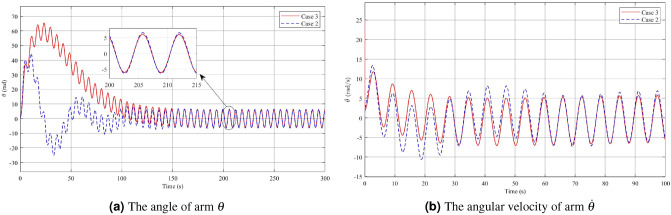
Angle and Angular velocity of the arm in the second case and third case.

**Figure 11:**
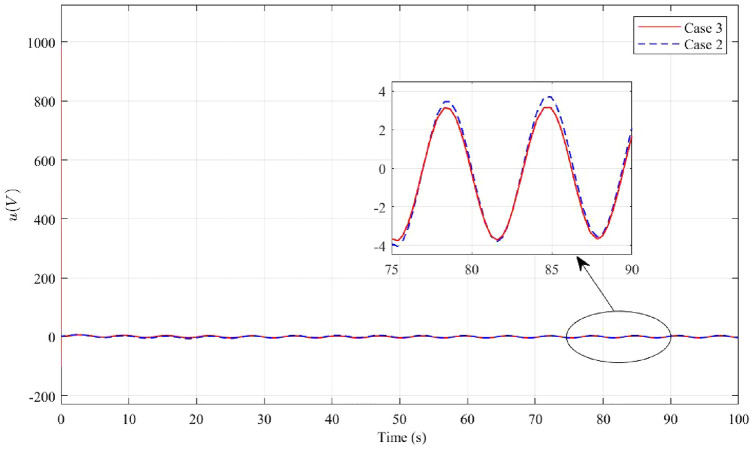
The voltage *u* in the second case and third case.

The simulation results comparing Case 2 and Case 3 demonstrate the effectiveness of incorporating both Theorem 4 and Theorem 2 to reduce the fluctuations in the pendulum angle, see Figs. 9, 10, 11. Figure 9a,b illustrate that the use of a controller with limited angle $\phi$ improves disturbance rejection performance. The maximum amplitude of the angle $\phi$ after reaching the steady state is significantly reduced to approximately 0.0056 rad, with an angular velocity of 0.0059 rad/s. However, during the initial phase, the pendulum angular velocity exhibits a more pronounced change, with a variation of around 23 rad/s compared to about 1 rad/s for a conventional disturbance rejection controller. This is attributed to the fact that the angle $\phi$ constrains the oscillation range of the angle $\phi$ to a relatively small around the equilibrium position. It is observed that when the initial deviation angle $\phi _0=\dfrac{\pi }{12}$ is significantly far from the active region’s maximum boundary value of approximately 0.008 rad, the controller needs to apply a quick and forceful initial action to bring the pendulum into the desired range of oscillations. From the analysis of Figs. 10a,b, and 11, a similar that can be observed as in Figs. 3a,b, and 4. When there is a large and rapid change in the pendulum angular velocity, it affects the values of the arm angle, arm angle velocity, and control voltage, causing them to increase significantly. However, it is important to note that both controllers reach a steady state at approximately the same time. In the initial moments of the voltage response, there are two distinct phases characterized by rapid variations, each occurring within a few milliseconds. The first phase involves an increase in voltage from 0 to 989.9V to bring the pendulum from $\phi _0$ to the stable oscillation region. In the second phase, the voltage is reduced from 989.9V to -102V to maintain the pendulum in a stationary position due to inertia. After stabilization, the voltage settles within the control range of -3.6V to 3.24V. Furthermore, upon observing the system after it has reached a stable state, it is evident that the operating voltage range of the controller in Case 3 (with limited angle $\phi$) is smaller compared to that of the conventional disturbance rejection controller in Case 2.

In order to maintain stability and minimize the impact of disturbances on the pendulum, the voltage must be adjusted to counteract these disturbances. In Case 3, the results indicate that a higher level of disturbance rejection performance requires a larger starting voltage value. However, in practical applications, most systems operate at low or medium voltage levels. Therefore, achieving an acceptable level of disturbance rejection can be considered a success. In the case 1, a significant finding is that the input voltage of the system is directly proportional to the initial deflection angle of the pendulum. Therefore, reducing the system voltage can be achieved by decreasing the initial angle of the pendulum. However, this will result in a smaller operating range for the pendulum angle. Designing a controller that simultaneously satisfies disturbance rejection and achieves an optimal starting voltage value is a challenging task. It should be noted that in practical scenarios, improving one aspect often involves a trade-off with another aspect.

**Figure 12:**
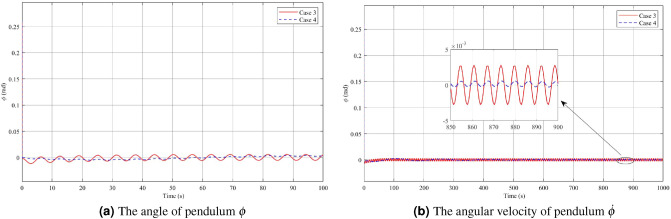
Angle and angular velocity of the pendulum in the third case and fourth case.

**Figure 13:**
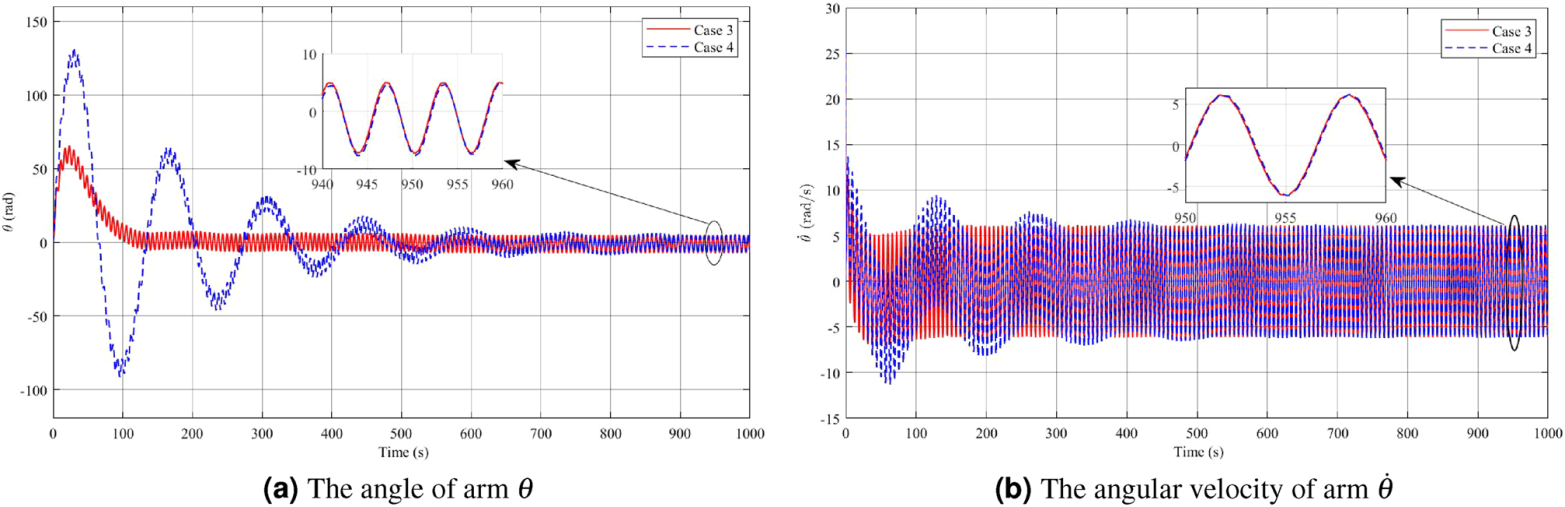
Angle and angular velocity of the arm in the third case and fourth case.

**Figure 14:**
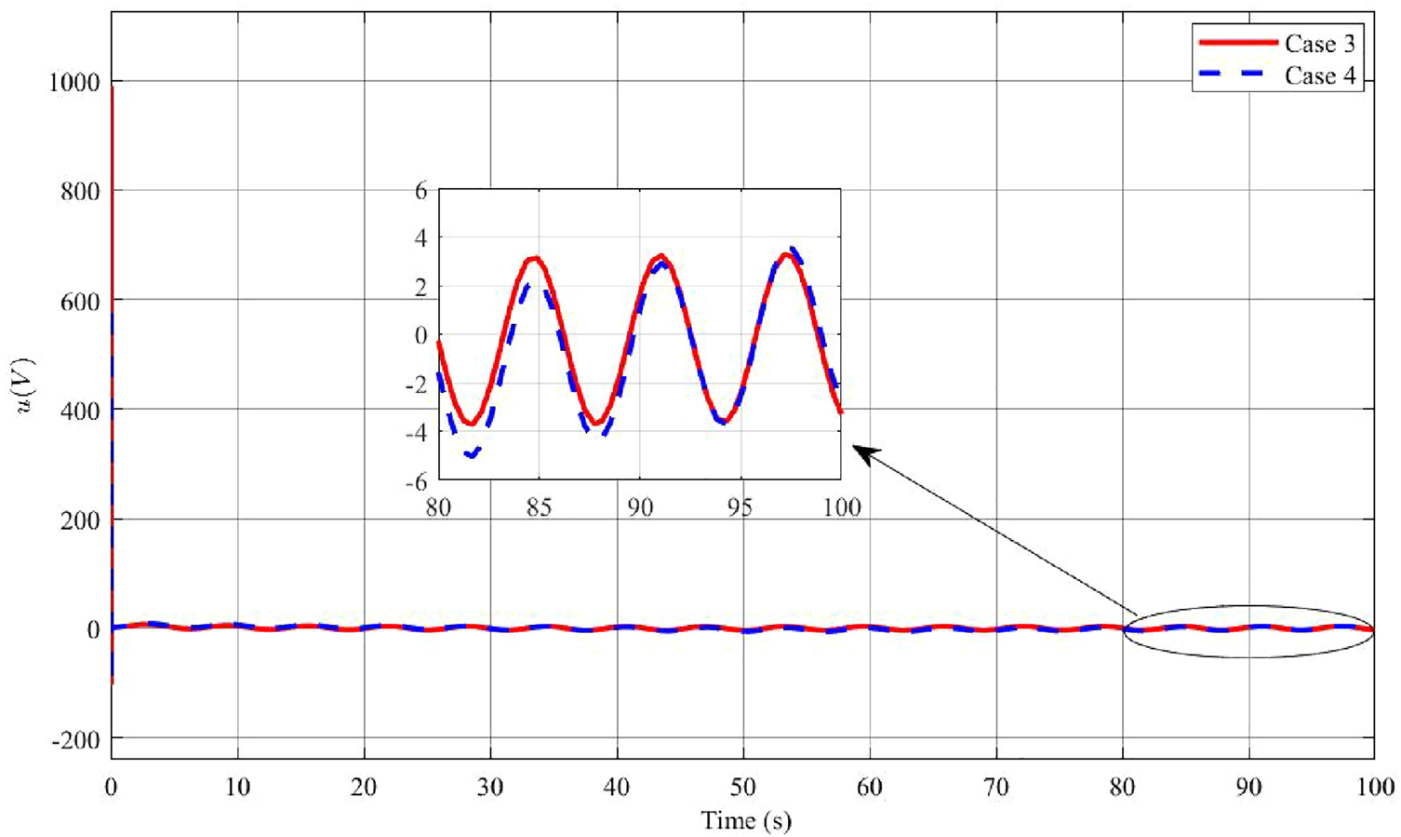
The voltage *u* in the third case and fourth case.

In Figs. 12, 13 and 14, the results of Case 4, where both disturbance rejection and input-output constraints are combined, are presented. By comparing Figs. 12a,b, it is evident that the controller in Case 4 yields significantly improved performance in terms of the deflection $\phi$ angle compared to Case 3. At steady state, the maximum amplitude of $\phi$ angle oscillation is reduced to 0.0004 rad in Case 4, whereas it is 0.0056 rad in Case 3. Furthermore, the change in $\dot{\phi }$ is also much smaller in Case 4 compared to Case 3. However, it should be noted that in Case 4, the oscillatory angle follows a second-degree oscillation pattern, requiring a relatively long time of approximately 800s to reach the steady state, as analyzed previously. Overall, the controller in Case 4 demonstrates improved performance in terms of reducing oscillations and achieving better stability, albeit with a longer convergence time. Upon examining Figs. 13a,b, and 14, it becomes evident that while both the arm angle and arm angular velocity exhibit fluctuations around the equilibrium position after the settling time, there is a noteworthy observation. The system’s settling time experiences the most significant alteration when employing the controller in Case 4. Once the steady state is reached, the amplitude of oscillation for the arm angle and arm angular velocity is nearly identical for both controllers. The large amplitude oscillation observed in the $\theta$ angle can be attributed to the prolonged stability time of the system. In Case 4, the starting voltage is reduced to 568.9V, causing a delay in the parameter settling time due to the significant impact of the reduced starting voltage. As mentioned earlier, a higher starting voltage value contributes to improved disturbance rejection efficiency. Therefore, when attempting to lower the starting voltage, the controller requires additional time to reach the steady state. Once the system has reached a steady state, the amplitude of voltage fluctuations in both Case 3 and Case 4 controllers is comparable.

To evaluate the disturbance rejection improvement of the controllers, this article employs a formula that compares the maximum oscillation amplitude of the $\phi$ angle between the case 1 without disturbance rejection and the others case 2, case 3 and case 4. The disturbance rejection improvement (*H*) is calculated using the following formula:

18 $$\begin{aligned} H = \dfrac{T_0 - T_1}{T_0} * 100\% \end{aligned}$$

where $T_0$ denotes the maximum oscillation amplitude of the $\phi$ angle in case 1, and $T_1$ represents the maximum oscillation amplitude of the $\phi$ angle in the others cases when the system reaches a steady state. This calculation provides a quantitative measure of the effectiveness of the controllers in reducing disturbance and improving system performance. The disturbance rejection improvement results for the different cases are as follows: in case 2, the disturbance rejection improvement *H* is 93.55$\%$. In case 3, the disturbance rejection improvement is 95.7$\%$. Lastly, in case 4, the disturbance rejection improvement is significantly higher at 99.67$\%$. These results demonstrate the effectiveness of the controllers in reducing disturbance and enhancing the stability and performance of the system.

The paper yields several notable findings. Firstly, the initial deflection angle of the pendulum showed direct proportionality to overshoot, transient time, and starting voltage. In the presence of disturbance, controllers with disturbance rejection can improve the pendulum angle and velocity, but require the compensation for disturbance effects on other aspects. These results enhance our understanding of system behavior under disturbance rejection and offer insights for designing controllers in practical applications.

## Conclusion

In conclusion, this study focused on the development and analysis of different control strategies for a RIP system. Through the utilization of various theorems and controllers, including disturbance rejection and input-output constraints, the performance and stability of the system were evaluated. The results showcased the effectiveness of the proposed controllers in mitigating disturbances and achieving desirable system behavior. Furthermore, the trade-offs between disturbance rejection, starting voltage, and settling time were explored, providing insights into the design and implementation of controllers for practical applications. Overall, the findings of this study contribute to the advancement of control techniques for RIP systems and highlight the importance of considering multiple factors when designing controllers to achieve optimal system performance. Future research may further investigate the application of these controllers in real-world scenarios and explore additional optimization strategies to enhance system stability and performance.

## Data Availability

The datasets used and/or analyzed during the current study are available from the corresponding author on reasonable request.
